# The effects of local socio-political events on group cohesion in online far-right communities

**DOI:** 10.1371/journal.pone.0230302

**Published:** 2020-03-30

**Authors:** Ana-Maria Bliuc, John M. Betts, Nicholas Faulkner, Matteo Vergani, Rui Jie Chow, Muhammad Iqbal, David Best

**Affiliations:** 1 Department of Psychology, University of Dundee, Dundee, United Kingdom; 2 Faculty of Information Technology, Monash University, Melbourne, Victoria, Australia; 3 Behaviour Works, Monash University, Melbourne, Victoria, Australia; 4 Alfred Deakin Institute for Citizenship and Globalisation, Deakin University, Melbourne, Victoria, Australia; 5 Institute for Sustainable Industries & Liveable Cities, Victoria University, Melbourne, Victoria, Australia; 6 Department of Criminology, University of Derby, Derby, United Kingdom; Universidade Estadual de Maringa, BRAZIL

## Abstract

In recent years, the reach and influence of far-right ideologies have been extended through online communities with devastating effects in the real world. In this research, we examine how far-right online communities can be empowered by socio-political events that are significant to them. Using over 14 years of data extracted from an Australian national sub-forum of a global online white supremacist community, we investigate whether the group cohesion of the community is affected by local race riots. Our analysis shows that the online community, not only became more cohesive after the riots, but was also reinvigorated by highly active new members who joined during the week of the riots or soon after. These changes were maintained over the longer-term, highlighting pervasive ramifications of the local socio-political context for this white supremacist community. Pre-registered analyses of data extracted from other white supremacist online communities (in South Africa and the United Kingdom) show similar effects on some of the indicators of group cohesion, but of reduced magnitude, and not as enduring as the effects found in the context of the Australian far-right online community.

## Introduction

Far-right political groups have been entering the mainstream of politics in many Western nations, arguably aided by a political climate dominated by mistrust, intergroup tensions, and increasing authoritarian attitudes [1–4]. More general support for the far-right movement, however, has been boosted by online communities of activists. Such communities are dangerous, not only because they undermine trust, create division, and increase intergroup tensions in society, but also because they provide isolated extremists with virtual communities of support. For example, Stormfront, one of the largest and most influential white supremacist online communities in the world—with over 300,000 registered members and more than one million visitors each month [5], has likely empowered ‘lone wolves’ such as the Norwegian right-wing extremist, Anders Breivik, who killed 77 people in Norway in 2011 [6] and who at the time of the killings was a registered member [7]. More recently, the perpetrator of the 2019 Christchurch mosques attacks in New Zealand responsible of 50 deaths, was at least partially radicalised online with social commentators making direct links between engagement with online far-right communities and the political violence perpetrated ‘in real life’ [8]. Given the growing influence that online far-right communities can wield in a technologically networked world [9, 10], we seek to investigate how such groups might become more cohesive, and therefore, more influential in society. In particular, we focus on examining how the internal cohesion of particular far-right online communities may be affected by offline socio-political events of significance to these communities.

## Theoretical background

### Group cohesion in offline groups and online communities

Research on small group dynamics shows that groups become more cohesive in the aftermath of specific events primarily because group members come to perceive these events as collective achievements or *group successes* [11–13]. Perceptions of group success may lead to a stronger sense of social identification with the group and thus increase group members’ perceptions of collective efficacy [14, 15]. Also, according to self-categorization theory [16–17], increased group cohesion can occur as a direct result of a specific collective identity being activated (or made salient) by contextual changes such as particular socio-political events driven by *intergroup conflict*. Intergroup conflict highlights self-categorizations in terms of ‘us versus them’ and socio-political events underpinned by such conflict can provide the group with clearer goals, a shared vision, and prescribe clear guidelines for behaviours in alignment with the group norms [18, 19]. Moreover, the specific conditions created by intergroup conflict may help opposing groups identify more clearly their respective group’s enemy and thus provide a highly specific basis for identity formation, increased identification with the group [20, 21], and unified action to achieve group goals. For example, the dynamics that may drive group cohesion within groups in conflict are well illustrated by clashes between protesters and counter-protesters in political demonstrations where markers of both group identity (what unifies the group) and outgroup differentiation (what sets the groups apart) are clearly displayed and strategically used. Such conflictual interactions tend to further accentuate the differences between ingroup and outgroup members, or in theoretical terms, increase the meta-contrast between the opposing groups [22]. The accentuation of group differences in a situation of intergroup conflict can, in turn, lead to stronger group identification and an increased sense of solidarity between group members on both sides [23].

In the current research, we seek to test whether the social psychological processes underpinning group cohesion (previously tested in small groups and different types of teams) apply to online far-right communities. Drawing on these theoretical points, we propose that socio-political events which are a) significant to online far-right communities, b) perceived as ingroup achievements, and c) driven by intergroup conflict, can strengthen these communities by increasing their internal cohesion.

Online communities can be seen as *psychological groups* in the sense that being a member of such groups is conducive to a psychological state of togetherness and belongingness even when members are not together in a physical space [17]. As with all social groups, online communities are based on shared social norms and values, and even more so than face-to-face groups, they are formed to enable and support the continuous consensualisation of attitudes and values. In this way, validation and intragroup influence, the two processes that directly affect the group structure, are achieved through mutual interaction [17].

If we think of group structure as a dynamic concept incorporating social interactions and relationships between group members within their group (intragroup processes), then the ‘digital footprint’ of members of an online far-right community represents a particularly rich data source. We consider several aspects of the intragroup dynamics as indicators of *group cohesion*, a socio-psychological construct first defined by Festinger in the 1950s to refer to those forces acting on group members to stay in the group [24]. Later definitions refer to group cohesion as a property derived from the strength of mutual positive relationships between group members [25] or as a dynamic process reflected in the tendency of a group to stick together in the pursuit of its goals [26]. In small group research, group cohesion has been related to levels of group performance [11, 12], while in studies of online communities, it has been associated with increased commitment and loyalty to these communities [27–29].

Classic research in social psychology treats an individual’s *attraction to the group* as the essential aspect of group cohesion [30]. However, conceptualisations of cohesion which predominantly rely on intragroup attraction are now seen as reductionist because they overlook other fundamental assumptions that underlie the nature of social groups [31]. More current conceptualisations treat group cohesion as a multidimensional construct which can be captured by both group members’ beliefs about *attraction to the group*, and levels of *group integration*. According to these definitions, attraction to the group refers to members’ perceptions about the capacity of the group to satisfy personal needs and objectives [26]. Thus, it encapsulates personal motivations and feelings about staying in the group. Group integration, on the other hand, refers to members’ closeness, bonding, and degree of unification within the group. The distinction between attraction to group and group integration represents the basis of an operational conceptualisation which was used to develop (widely used) self-report measures of group cohesion in sports teams, but with a broader applicability to other types of groups [26].

In our research, we adopt and build on these operational conceptualisations to investigate variations in levels of group cohesion in several national sub-forums of the global online white supremacist community Stormfront.org. To capture group cohesion in this context, we examine indicators derived from conceptualisations of group cohesion as encompassing both *attraction to the group* and *group interaction*. Self-report measures of group cohesion applied to small groups (that is, The Group Environment Questionnaire, 26), include sub-scales of attraction to the group and group interaction which refer to both the group (social dimensions) and the task (instrumental dimensions). Because our study groups are online communities where interactions between group members occur via online exchanges and there are no set group tasks to be accomplished (thus differing in significant ways from small teams in sporting or organisational contexts), we only use those indicators which are expected to capture the social dimensions of group cohesion. As our study focuses on an online community, we use members’ levels of individual connections to other group members to capture *attraction to the group* (inter-member attraction). Based on its operational definition, we consider group integration as being indicated by members’ levels of bonding and unification of the group, and we consider engagement of group members with the online community as encapsulating closeness between members (i.e., developing familiarity or a desire to increased interaction). Thus, the indicators of group integration that we use are:

- *Bonding*, as connections between the most committed members of the group or the most active members on the forum;
- *Unification*, as the number of members that interact directly under a common thread (that is, members discussing the same topic reflecting a consolidation of ideas within the online community); and
- *Engagement*, as increased levels of social interaction by members; that is, less active members becoming more active.

## The current research

Recent research [32] identified racist riots occurring in Sydney in 2005 (known as the Cronulla Riots) as a highly significant socio-political event for the online community Stormfront Downunder. The riots were found to be associated with changes in the social identity content of the online community. Detailed analysis of the language used by the members of the online community, demonstrated that norms, emotions, and key concerns of the group significantly changed in the aftermath of the riots suggesting a sharpening and crystallisation of the collective identity of the community towards becoming more blatantly anti-Muslim. In the current research, we first seek to establish whether the riots had any longer-term effects on the internal cohesion of the same online community, Stormfront Downunder, and then test whether these effects can be found in other similar online communities (in relation to offline events significant to them).

### Racist riots in Sydney as a significant event for the far-right online community Stormfront Downunder

The Cronulla Riots occurred in the Sydney beach suburb of Cronulla on December 11, 2005 in the form of violent demonstrations against Australians of Muslim backgrounds. The riots were sparked by an earlier altercation between several Middle Eastern beachgoers and Anglo-Saxon lifesavers. What initially started as a protest ‘to reclaim the beaches’, quickly escalated to violence when about 40 people attacked a man of Middle Eastern appearance. The violence during the riots resulted in more than 25 injured people and destruction to property. Importantly, members of local white supremacist groups and radical right-wing political parties were present at the riots and seen distributing pamphlets [33, 34]. Going beyond injury to people and property, the events have a deeper significance in the Australian context, representing the beginning of intense debates in the public arena about the meaning of being Australian in today’s multicultural society [32]. A key aspect in the context of this research is that the riots represent an unprecedented blatant and violent public expression of anti-Muslim sentiment in modern Australia.

Using the Cronulla Riots as the reference point for the online community Stormfront Downunder, we first sought to establish whether the increase in the levels of activity in the sub-forum after the riots was statistically significant. Second, we tested whether group cohesion significantly increased after the riots and whether its indicators remained high in the following months and years. Specifically, we predicted increases in the indicators of group cohesion after the riots as follows:

1. *Attraction of the individual to the group*: we expected to find more individual connections between group members, and those levels to be maintained in the longer term.
2. *Bonding*: we expected to find more connections within the group between the highly active members (both old and new members).
3. *Unification*: we expected fragmented conversations to become unified under shared threads–thus more members would interact via fewer threads, rather than interacting under many smaller threads.
4. *Engagement*: we expected inactive members to become active (re-engagement), and new members to have comparable or higher levels of activity than existing active members.

### Identifying significant events for online far-right communities in other contexts (South Africa and Britain)

To determine whether the same effects can be found in other national contexts in similar online communities, we analysed data from Stormfront South Africa and Stormfront Britain. Given that our initial study on Stormfront Downunder is a natural experiment conducted outside the laboratory, we did not expect that data from different socio-political and cultural contexts, related to different events, and from different populations to provide exact replications of the findings. Rather, these studies are closer to ‘constructive replications’ [35] where several new elements are introduced and a similar (but not identical) effect is expected. These analyses were conducted before and after significant events in each of the respective national contexts. We identified two possible significant local socio-political events in the context of the far-right movement in South Africa and respectively the UK by applying several criteria. We established these criteria based on the theoretical points that we applied in our analysis of the Cronulla Riots as a significant local event for the far-right movement in Australia. These criteria are: a) there is *ideological alignment* between the drivers of the event/collective action and the online community, whereby the event involves some form of collective action by a group that is ideologically aligned to the online community; b) there is some degree of *intergroup conflict*, that is, the event is underpinned by intergroup conflict between the group ideologically aligned to the online community and an outgroup; c) the *collective goals* driving the group’s action ‘in real life’ are also *shared* by the online community (in the Cronulla Riots the action was about ‘reclaiming the beach’ from a cultural outgroup; similar events may be about claiming of reclaiming of political power, rights, status, etc.); and d) there is a sense of group achievement brought by the event or *perceived group success*, that is, the outcome of collective action has a positive connotation for the online community (in the case of the Cronulla Riots, the sense of achievement was likely derived from the perception that the riots represented an important act of rebellion against a system promoting multiculturalism with the sense of importance being enhanced by the significant media attention). Using these criteria, we identified the following socio-political events in the context of South Africa and the UK (based on the assessment that they meet as meeting at least one of the criteria):

1. *Protest against the name change from Pretoria to Tshwane in May 2005* (Stormfront South Africa). In this event, thousands of protesters marched against the decision to rename the city (Tshwane in Setswana language means ‘we are the same’). The local event represents an attempt to maintain or *re-claim status* by the white community, a goal which is ideologically consistent with the South African far-right movement. The action was driven by intergroup conflict between supporters and opponents of the change, but unlike the case of the Cronulla Riots, there was no violence between the groups. Also, the collective action did not result in a reversing of the decision to change the name back to Pretoria, so it is plausible that the sense of group achievement was reduced compared to the Cronulla Riots.
2. *The far-right gaining political representation in the UK and the Assembly of London elections in May 2008* (Stormfront Britain). In this event, the far-right British National Party (BNP) had an historic win, gaining 30 seats in the local elections and for the first-time gaining representation in the Assembly of London. This event represents a successful *claiming of political power and legitimacy* and therefore it is likely that it elicited a sense of group achievement in the Stormfront Britain online community. However, (as a point of difference from the Cronulla Riots) the achievement was brought about via a democratic process where intergroup competition rather than hostile intergroup conflict was salient.

## Method

We conducted a natural experiment (using a quasi-experimental paradigm) where, the experimental manipulation was introduced by the Cronulla Riots as significant socio-political event for the far-right community in Australia, and thus to the online community Stormfront Downunder. For the other national contexts, as significant events for Stormfront South Africa and Stormfront Britain online communities, we used the 2005 Tshwane protest and respectively the 2008 UK election.

### Statement of ethics

This research was approved by the Monash University Research Ethics Committee, Project ID: 8420.

### Data collection

We analysed data from three Stormfront sub-forums: Downunder (Australia) for our main study, and South Africa and Britain (UK) to see if the effects found in the main study can be replicated. For each of the three sub-forums, we examined the following metadata: user identifier number, thread identifier number, as well as the time and date of each post, and word count for each post. Other content, such as graphics, video or audio was not retrieved. For this study, we analysed all posts made to the general discussion section of each of the sub-forums over the periods specified. These were: Stormfront Downunder, https://www.stormfront.org/forum/f116/ from 13/09/2001 to 06/05/2015, (75,795 posts, 2,520 anonymous). Stormfront South Africa, https://www.stormfront.org/forum/f113/ from 13/09/2001 to 06/05/2015, (49,436 posts, 870 anonymous), Stormfront Britain, https://www.stormfront.org/forum/f92/ from 13/09/2001 to 06/05/2015 (210,117 posts, 1,495 anonymous).

In Stormfront Downunder, we first examined variations in levels of online activity and then variations in indicators of group cohesion to see whether there were significant changes in relation to the riots. Specifically, we examined indicators of activity and connections between members, weekly, over the six months before, during, and after the riots. These were: the number of posts made (overall activity), connections per member (individual attraction to group), connections per member for the 50 most active members (bonding between most committed group members), posts per thread (unification of members interacting under the same threads), and the number of active members (engagement). For this analysis, posts were grouped into weeks (7 days) centred on the first day of the Cronulla Riots, December 11^th^, 2005. The week of the riots was denoted as Week 0. Posts include both users’ own initial posts and replies to posts by other users. Raw (typically count) data was used to test the hypotheses without any seasonal adjustment of activity time series. To test the sensitivity of our analysis to the choice of a 7-day week, the same analysis was performed with week lengths of 3, 14 and 28 days, which showed that the substantive conclusions of our analysis were unaffected.

**Fig 1:**
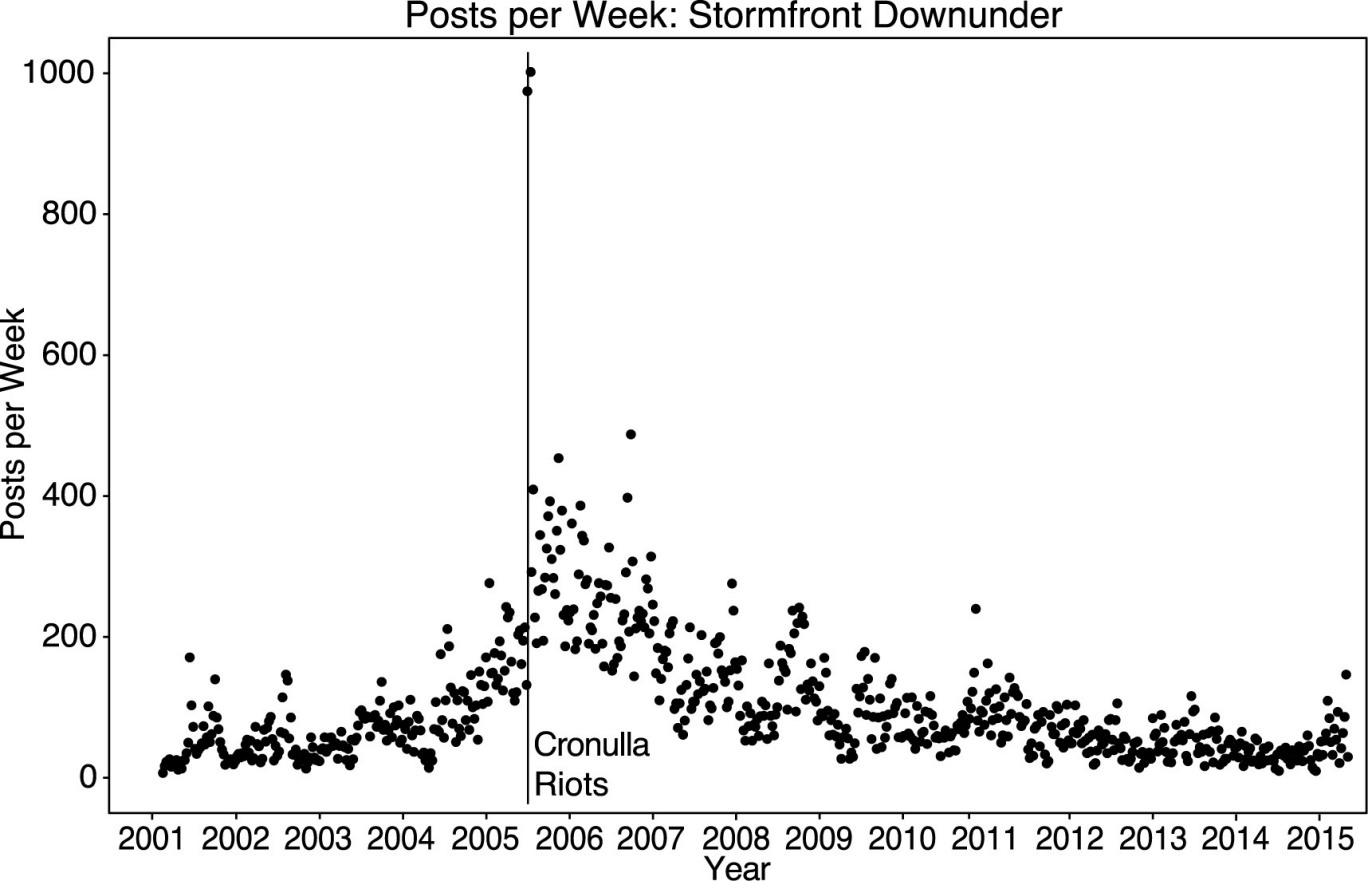
Number of posts per week to the Stormfront Downunder sub-forum 2001 to 2015. Increased posting activity is observed around the Cronulla Riots in Sydney, December 2005.

All posts were included in the analysis of activity, such as the number of posts per week (Fig 1), and the number of posts per thread. The distribution of word counts in all sub forums was positively skewed, therefore the hypothesis tests report results for log-transformed data, summary statistics, however, report raw data. Anonymous posts were omitted from the social network analysis and count of active members as these could not be attributed to specific authors. For Stormfront Downunder, anonymous posts comprised only 3.3% of the cohort and did not significantly differ in average word count (*M* = 104.64, *SD* = 166.26) for members, and (*M* = 110.18, *SD* = 198.33) for anonymous posts as shown by the two-sided t-test, *t*(2686) = 0.32, *p* = 0.747, *d* = 0.03.

For Stormfront South Africa, anonymous posts comprised 1.8% of posts. There was a significant difference in word count, due in part to the presence of several extremely long posts by members (*M* = 213.66, *SD* = 374.21), and (*M* = 127.04, *SD* = 283.60) for anonymous posts, (two-sided) *t*(1068) = -12.31, *p* << 0.001, *d* = -0.23. Stormfront Britain contained 0.7% anonymous posts with a small but statistically significant difference in post length, (*M* = 99.78, *SD* = 178.36) for member posts, and (*M* = 111.57, *SD* = 145.34) for anonymous, (two-sided) *t*(5588) = 10.84, *p* << 0.001, *d* = 0.07. However, for both South Africa and Britain sub-forums, these anonymous posts only comprise a very small proportion of each cohort, therefore we conclude that the omission of anonymous posts does not unduly bias these analyses.

Further information on each of the figures, hypothesis tests, as well as the R scripts used to analyse the data from all three sub-forums are presented in the S1 Data. The analyses of Stormfront South Africa and Stormfront Britain data were pre-registered at AsPredicted.com (public pre-registration: https://aspredicted.org/9j8gi.pdf).

## Results

### Differences in the activity of the online communities before and after the events

There is a significant increase in Stormfront Downunder activity during the period immediately before, during, and in the aftermath of the riots. Fig 1 shows the level of online activity between 2001 and 2018 as the number of posts made by group members each week. The peak in posts late in 2005 coincides with the riots in early December 2005. Around the time of the riots, an immediate increase in online activity can be observed—for example, during the six months (26 weeks) immediately before the riots, members made, on average, 187.88 posts per week (*SD* = 52.06). Over the 6 months following the riots (excluding the two weeks of the riots) members made 329.85 posts per week on average (*SD* = 81.68), representing a significant increase as confirmed by a one-sided t-test, *t*(42) = 7.47, *p* < 0.0001, *d* = 1.44. As Fig 1 shows, these high levels of activity were maintained over the whole of 2006, and gradually dissipated over subsequent years. Because the number of posts was significantly greater over the period immediately before and after the riots than at any other time in the forum’s history (even when controlling for seasonal trends), we used the riots as the reference point to assess changes in group cohesion of the online community.

**Fig 2:**
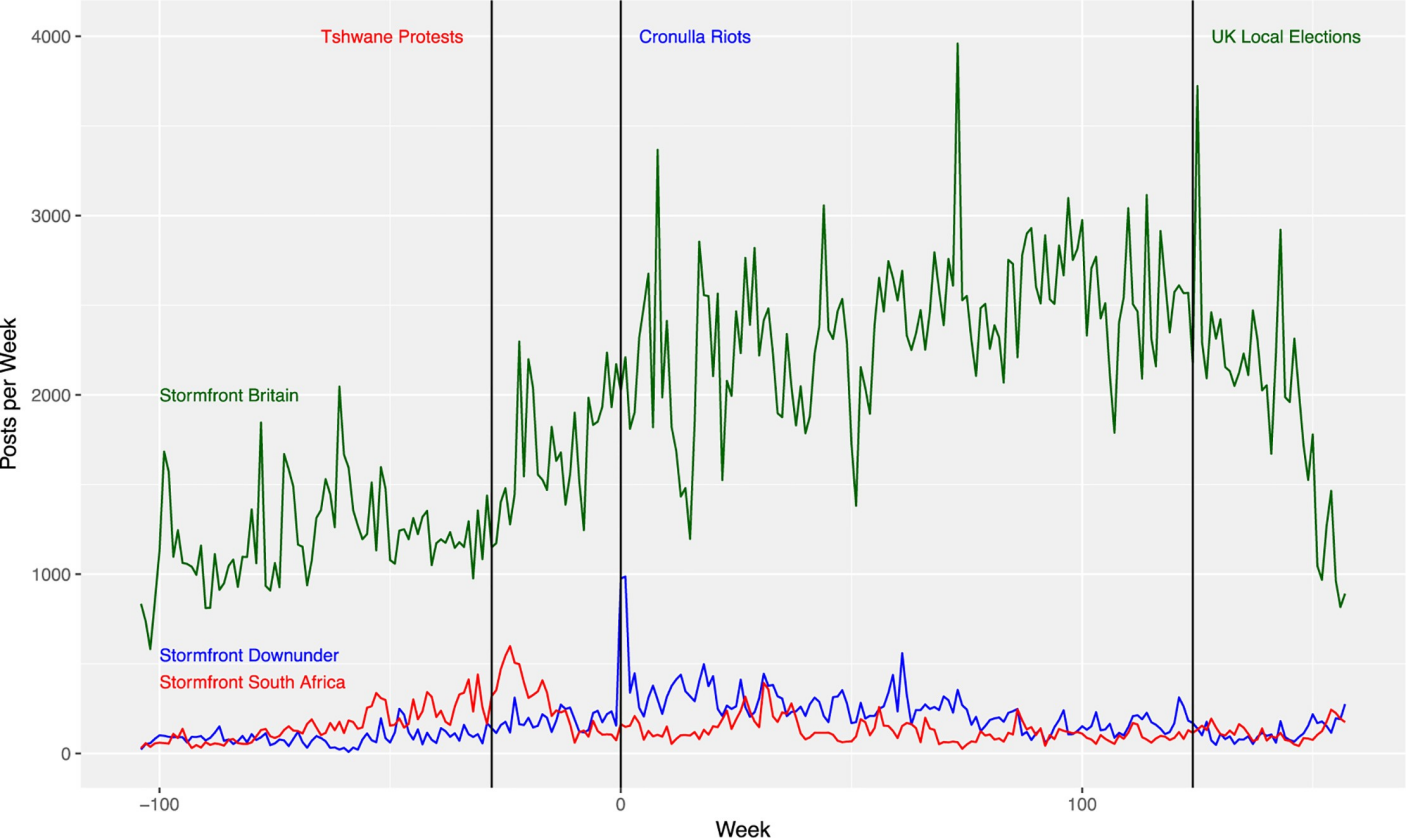
The number of posts per week in the Stormfront Downunder, Stormfront South Africa and Stormfront Britain sub-forums. The significant event relative to each community is indicated at the time of the event.

For Stormfront South Africa, there is not a significant change in levels of activity, as indicated by posts per week, from 6 months before (*M* = 258.38, *SD* = 80.65) to 6 months after (*M* = 277.04, *SD* = 159.22) the Tshwane Protests, (one-sided) *t*(37) = 0.54, *p* = 0.30, *d* = 0.15. However, as Fig 2 shows, there is a spike in activity post-Tshwane Protests. Performing the same hypothesis test, but over a shorter period (3 months = 13 weeks) shows that there is a significant increase in forum activity after the Tshwane Protests, from an average number of posts per week during the 3 months prior to the riots of 285.23 (*SD* = 86.56) to an average of 408.15 (*SD* = 109.63) over the 3 months post riots, (one-sided) *t*(23) = 3.17, *p* = 0.002, *d* = 1.06. For Stormfront Britain, there is a spike in forum activity around the time of the UK elections. However, no significant changes are detected over the intervals 6 months pre (*M* = 2539.96, *SD* = 309.35) and post (*M* = 2092.69, *SD* = 356.52) elections, (one-sided) *t*(49) = -4.83, *p* = 1, *d* = -1.12, or at 3 months pre (*M* = 2527.92, *SD* = 277.47) and post (*M* = 2243.39, *SD* = 145.67) elections, (one-sided) *t*(18) = -3.27, *p* = 1, *d* = -1.08.

### Attraction of the individual to the group

**Fig 3:**
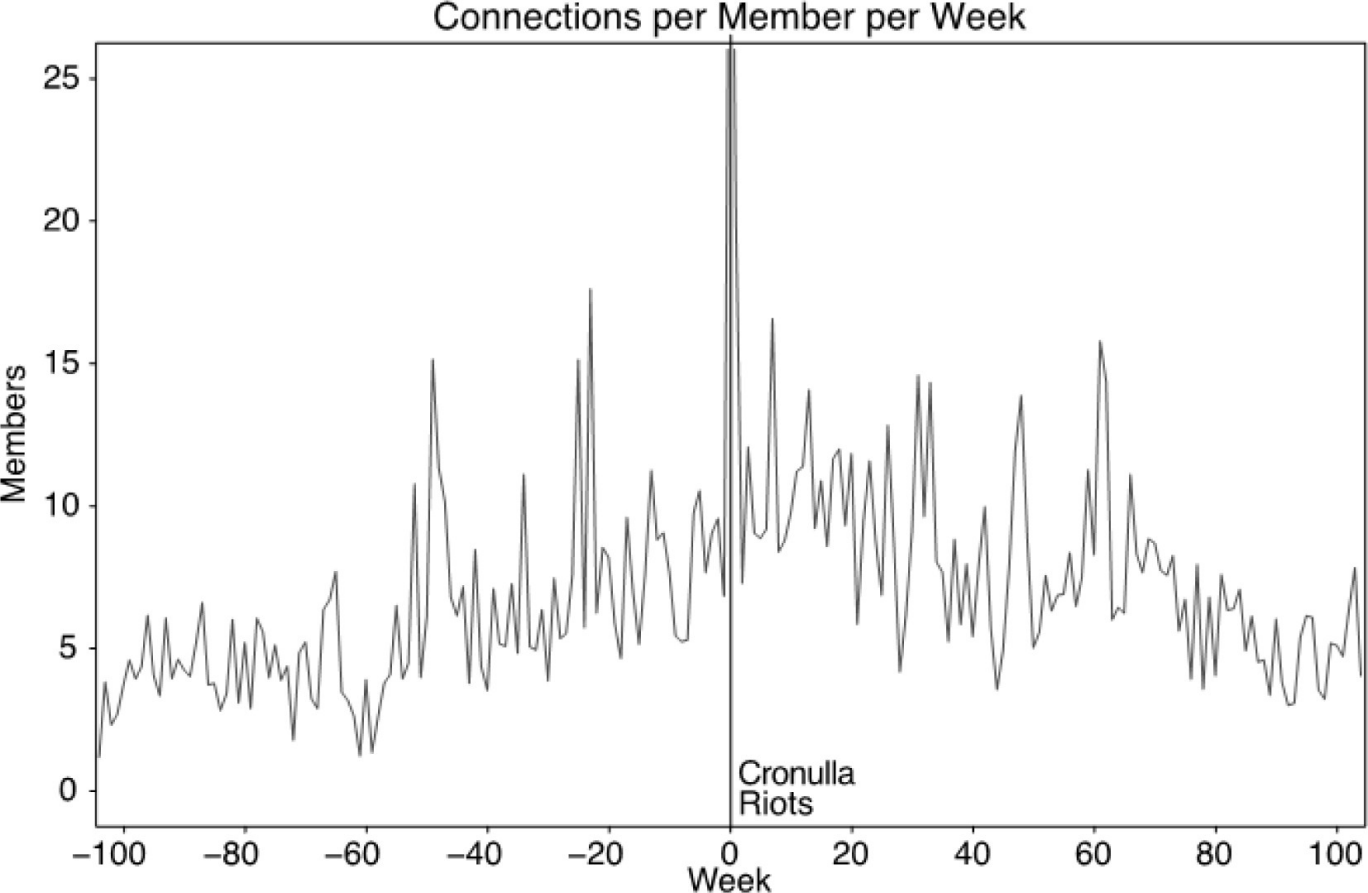
The average number of social network connections in Stormfront Downunder per member, weekly, from two-years prior to two-years post Cronulla Riots.

Individual attraction was captured by the number of connections between members before and after the riots. This social network was constructed using a two-mode concept [36], whereby members were considered linked if each had posted to the same thread within a specified time interval, set as one week for our analysis. Fig 3 shows the average number of connections between all members of the Stormfront Downunder, calculated weekly, during the two years before the riots and two years after the riots. The average number of connections between members increased significantly after the riots, from an average of 8.50 (*SD* = 3.00) connections per member per week during the 6 months before the riots, to 10.39 (*SD* = 2.33) connections per member per week during the 6 months after the riots, (one-sided) *t*(47) = 2.53, *p* = 0.007, *d* = 0.67.

In Stormfront South Africa, there is no significant change in the number of connections between members over the 6 months pre (*M* = 8.05, *SD* = 2.79) and post (*M* = 7.31, *SD* = 3.85) Tshwane Protests, (one-sided) *t*(45) = -0.79, *p* = 0.78, *d* = -0.22. However, over the shorter term (3 months), there is an increase in social connections pre (*M* = 8.32, *SD* = 2.50) and post (*M* = 10.01, *SD* = 3.15) Tshwane protests, (one-sided) *t*(22) = 1.51, *p* = 0.07, *d* = 0.58. In Stormfront Britain, there is no significant change in the number of connections between members pre- and post-elections over the longer term (6 months) pre (*M* = 29.17, *SD* = 5.14) and post (*M* = 23.99, *SD* = 4.77), (one-sided) *t*(49) = -3.77, *p* = 0.99, *d* = -0.93, or shorter term (3 months) pre (*M* = 27.77, *SD* = 4.32) and post (*M* = 25.26, *SD* = 3.15), (one-sided) *t*(22) = -1.68, *p* = 0.95, *d* = -0.64.

### Bonding between online community members–reinvigoration after the events

**Fig 4:**
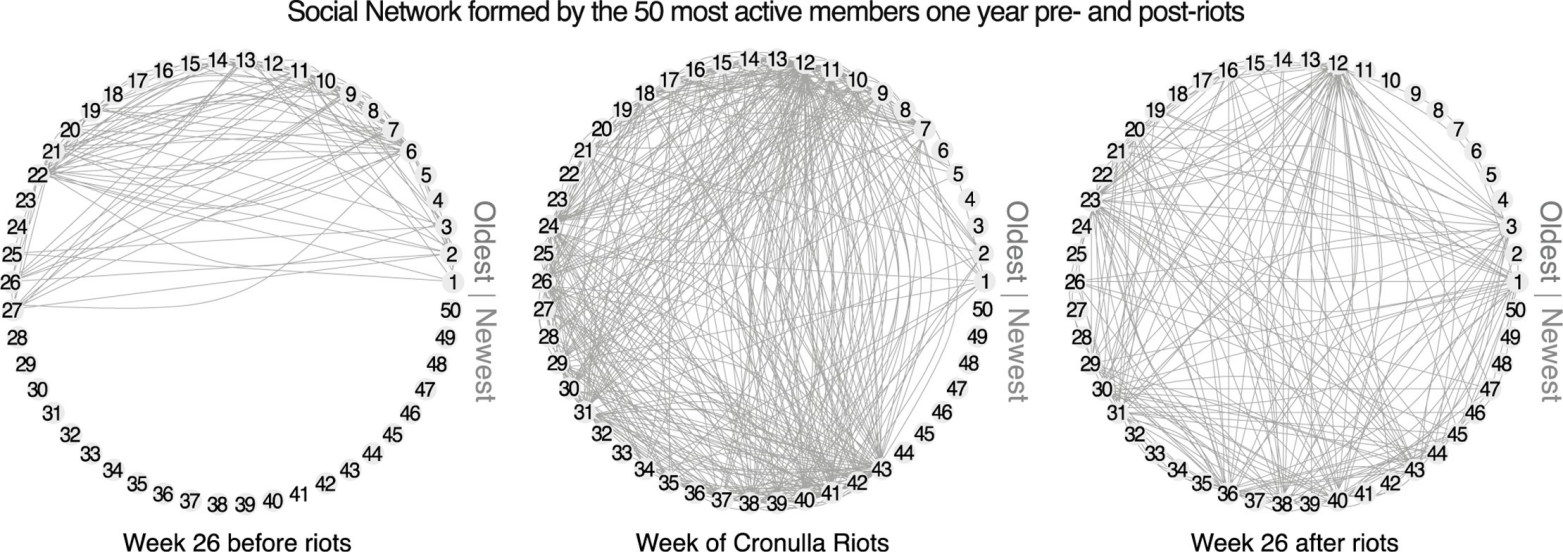
Snapshots of the social networks in Stormfront Downunder formed at 6 months (during Week 26) pre- and post-Cronulla Riots, and during the week of the riots by the 50 most active members over one-year prior to one-year post riots.

Bonding between the members of the online communities was measured by members’ posting activity and the creation of connections between existing active members and new members. We expected to find more connections in the social network between the most committed members of the online community. To examine this phenomenon, the social network formed by the 50 most active members over the period starting one year before the riots and ending one year after the riots was constructed at weekly intervals. In Stormfront Downunder, the reinvigoration of the online community after the riots due to the bonding of old and new members is evident in Fig 4, which shows snapshots of the social network formed at three points in time: before, during and after the riots. Each vertex represents a member of the online community, shown in order by the date of their first post to the forum (thus Member 1 is the longest standing member while Member 50 is the most recent). As Fig 4 illustrates, at 6 months before the riots, existing members were moderately well-connected. Among all members of the social network there was an average of 15.59 (*SD* = 10.86) connections per member. During the week of the riots the network had become highly connected, with an average of 106.10 (*SD* = 75.43) connections per member over the whole network. Importantly, new members were communicating with long-standing members. Finally, at 6 months after the riots, a high level of communication between members had been maintained, with an average of 26.09 (*SD* = 26.45) connections per member. The connections between older and newer members were preserved, which shows that new members were contributing to the renewed activity in the group.

**Fig 5:**
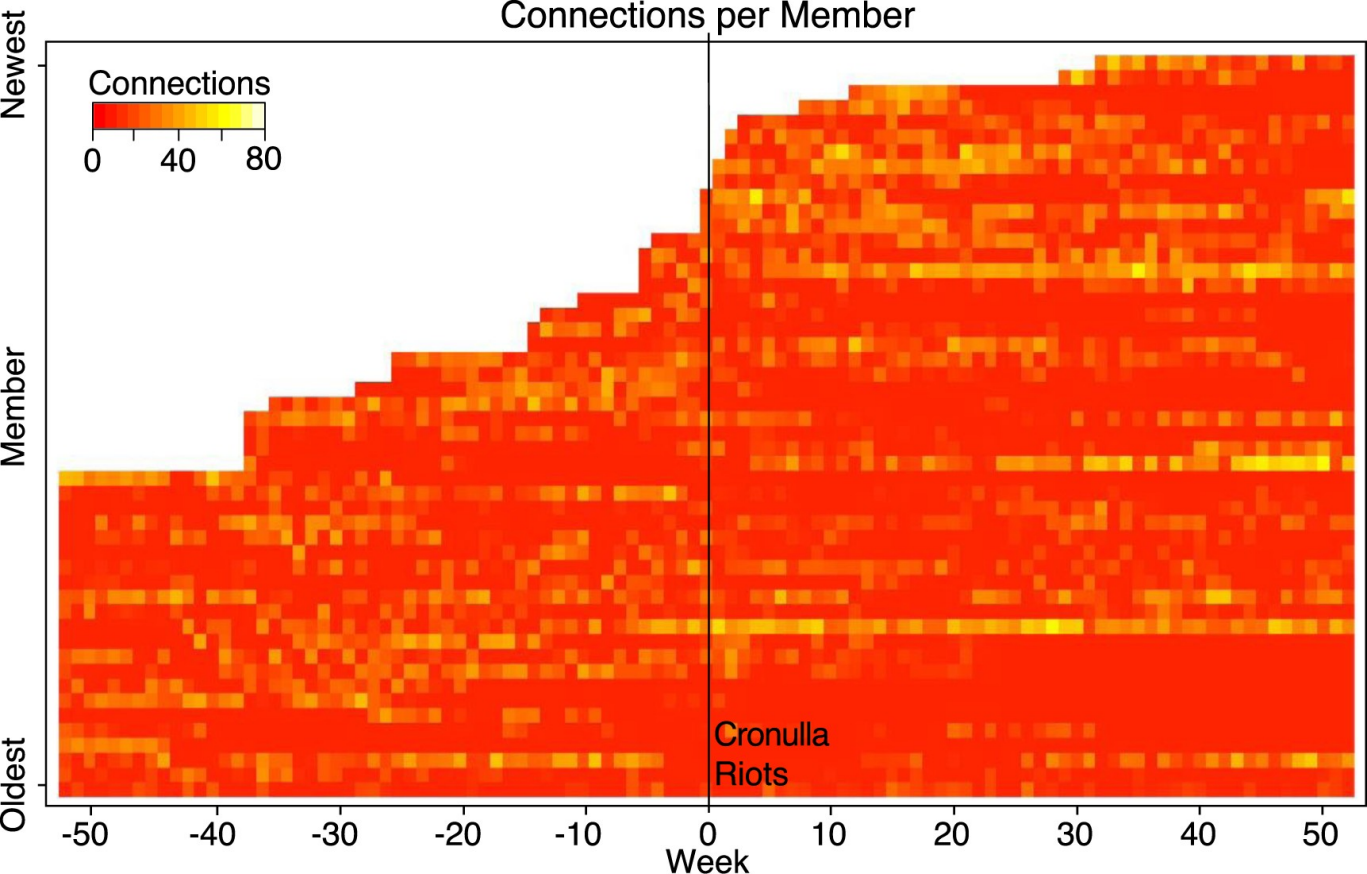
Heatmap showing the connections between the 50 most active members weekly, from two-years prior, to two-years post Cronulla Riots.

The high level of connectivity among new members joining around the time of the riots and soon after (suggesting reinvigoration of the group) is also shown in Fig 5. The heat map shows the number of each member’s network connections for each week from one year (52 weeks) before, to one year after the riots. New members were the most active members over the period analysed, having a high level of initial activity, shown as lighter areas on the heat map. By contrast, the longest standing members who joined the group before the riots did not show the same level of high initial activity and connectivity.

A small group of active, well-connected, new members joined Stormfront South Africa around the time of the Tshwane Protests, and remained active and connected with longer standing members at 13 weeks (S1 Fig in the S1 Data). However, reinvigoration of Stormfront Britain corresponding to the UK Elections is not evident (S2 Fig in the S1 Data).

### Unification in the online communities

**Fig 6:**
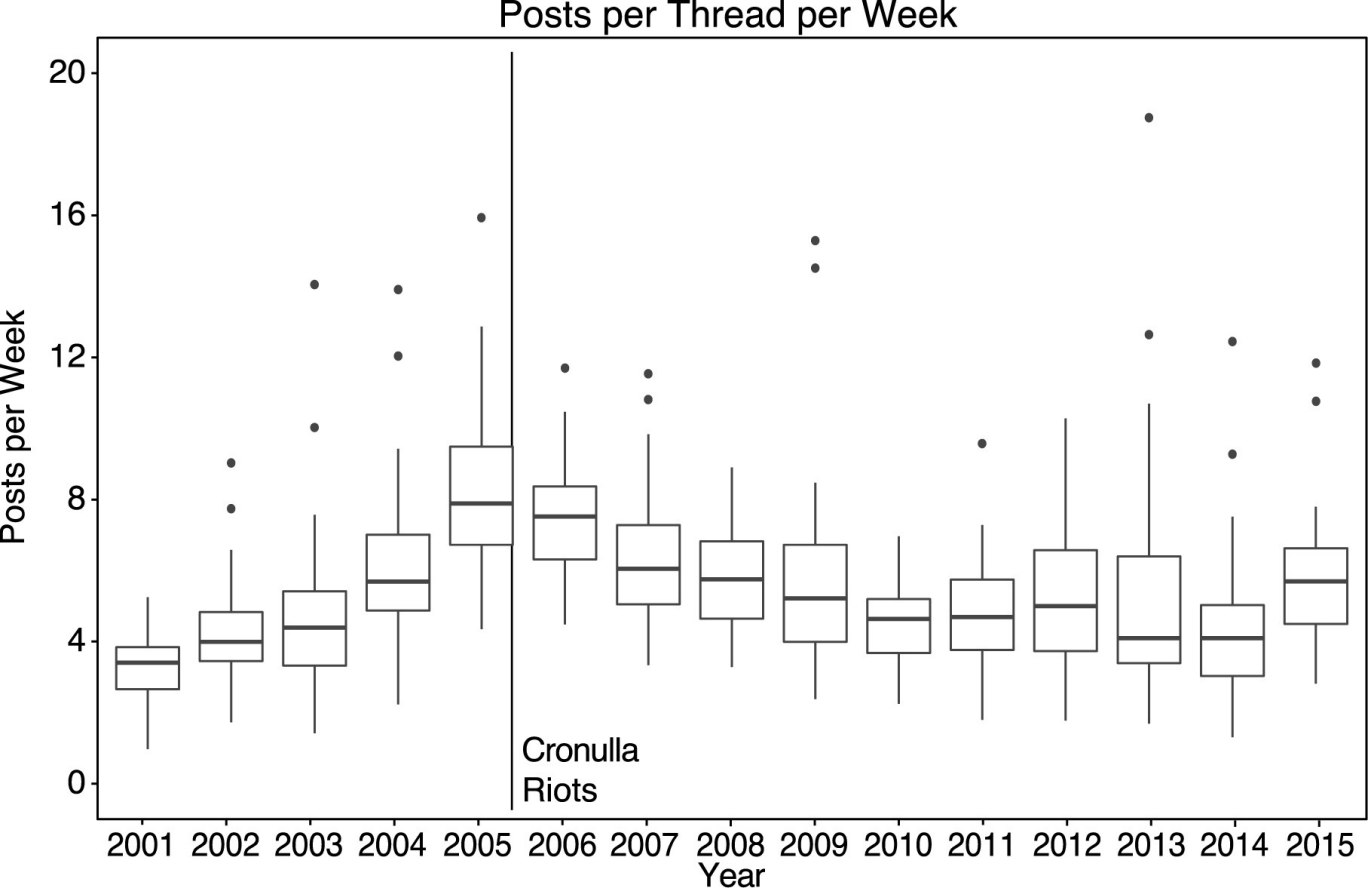
Posts per thread per week for all members of Stormfront Downunder, 2001 to 2015.

We predicted that after the event, unification would be reflected in fragmented conversations converging under shared threads–thus more members interacting more frequently through fewer threads. In Stormfront Downunder, our analysis shows that although the number of posts was greater immediately before and after the riots than at any other time in the forum’s history, the increased participation by members resulted in a greater cohesion of the group, rather than fragmentation. We used the number of participants posting to the same thread each week, *posts per thread*, as an indicator of group unification. Fig 6 shows that group unification was greatest close to the riots and preserved over the longer term. Using the period from 2001 to 2004 as a baseline, having an average of 5.04 (*SD* = 3.32) *posts per thread*, the increase in unification was significant (*p <* 0.01), for a one-sided t-test over the years 2005 to 2008 (S1 Data in S1 Table).

Analysis over the same time period shows that a similar effect, but over a longer duration, is evident for Stormfront South Africa in the years following the Tshwane Protests (S3 Fig in the S1 Data, S2 Table). Comparison of posts per thread by in the years 2008 to 2013 with those prior (years 2004–2007) shows a significant increase in 2008, the year of the UK Elections, and corresponding with a spike in sub-forum activity (S4 Fig in the S1 Data, S3 Table). However, this increase is not maintained in subsequent years.

### Engagement within the online communities

**Fig 7:**
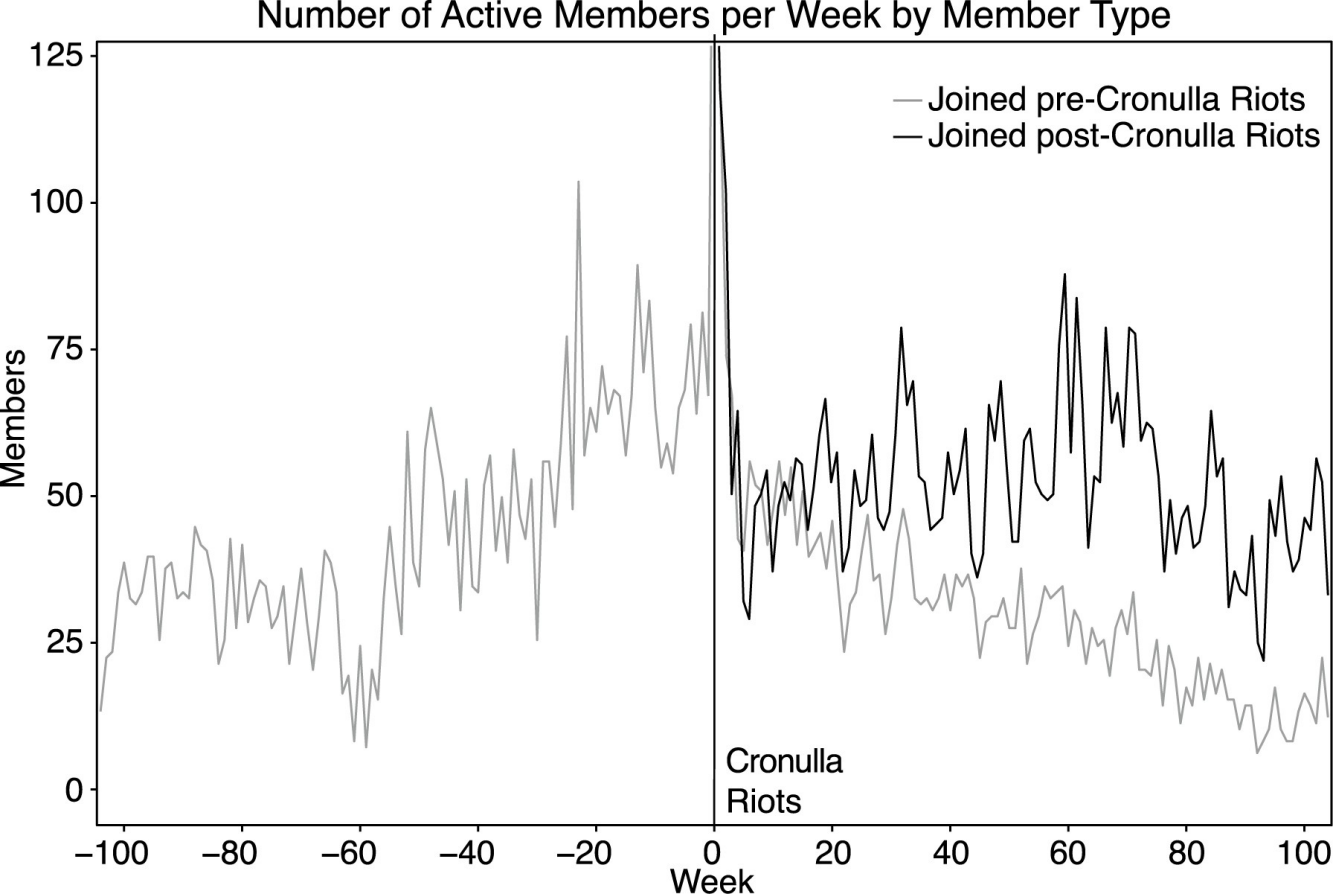
Number of active members per week from two-years prior, to two-years post Cronulla Riots, by whether the member joined pre- or post-riots.

We examined variations in levels of engagement of the online community as reflected by levels of activity by newer members, compared with those of longer standing. Fig 7 shows the number of Stormfront Downunder members participating in the online community, weekly, from two years before the riots until two years after the riots. Members were deemed to be participating if they made at least one post during any given week. Members who joined the group at any time before the riots were classified as *existing* members. *New* members were those who joined in the week of the riots or after. As illustrated in Fig 7, the level of activity of existing members declined after the riots. New members were driving the activity of the online community after the riots and the activity of these new members remained at a similar level even two years after the riots.

In Stormfront South Africa, a similar effect, but to a smaller degree, can be observed post Tshwane Protests (S5 Fig in the S1 Data). There is no significant increase in new members joining Stormfront Britain immediately post UK Elections (S6 Fig in the S1 Data), with the gradual increase in new members and decline in older members being more reflective of natural attrition and renewal within the sub-forum.

## Discussion and conclusion

Our findings provide evidence on how significant socio-political events ‘in real life’ can unite and reinvigorate particular white supremacist online communities. In the case of Stormfront Downunder, local race riots in Sydney had strong positive and long-lasting effects on the internal cohesion of the online community–after the riots, the community becomes more cohesive. In particular, the online community becomes more connected and reinvigorated primarily because, during and shortly after the riots, a) it attracted highly active and group-minded new members, and b) the frequency of interactions between the members increased. Regarding members’ engagement with the online community, we found that existing members, regardless of their previous levels of participation, became less active after the riots. By contrast, new members were driving the activity in the online community after the riots. The pattern of interactions between the most active members shows that during and after the riots, bonding increased, not only between existing members, but also between existing and new members. This latter type of bonding is particularly significant because it suggests exchanges and knowledge transfer that may drive changes in the collective identity of the group (that is, what members collectively stand for, their shared goals, values, and norms). This finding is consistent with previous research based on textual analysis of post content showing that this particular online community did indeed change after the riots–the group norms became more inclusive of ideologically similar others, but more exclusive of ethnic and religious outgroups; importantly, a stronger and clearer anti-Muslim stance was adopted by the group [30].

In the aftermath of the riots, the members of Stormfront Downunder communicated more with each other–that is, individual attraction to the group, as reflected in the average number of connections between group members, peaked during the week of the riots and remained elevated for a long period after the riots. The group also became highly unified after the riots when, more people interacted under fewer discussion threads. Thus, rather than members talking about many different things, there were more members collectively discussing the same issues. This dynamic suggests that a shared understanding of matters relevant to the group might may have increased consensualisation of group’s norms and values [37]. It implies that the legacy of the riots is group reinvigoration (thus going beyond increased group cohesion *per se*), possibly due to the attraction and retention of new highly active and group-minded members who are therefore more likely to maintain cohesion in the long-term. It is plausible that some existing and new members of the online community directly participated in the riots. For these members, their direct contribution to the collective action undertaken in the pursuit of shared group goals (based on beliefs in line with a white supremacist ideology) could have led to an even more heightened sense of achievement on the group’s behalf–hence their increased efficacy within the group long after the riots.

Our analysis demonstrates that, in the case of the online community Stormfront Downunder, significant local socio-political events can have strengthening and potentially transformative effects. These findings are consistent with theoretical propositions that emphasise the power of conflictual interactions between groups to shape and re-shape collective identities; that is, these interactions enable ‘newly empowered’ definitions of collective selfhood to emerge [38–40]. In the case of the Cronulla Riots, their evident direct and immediate effects are localised at the level of the Australian community and mostly consist of opening a national debate on national identity and nationalism.

However, the analyses of data from far-right online communities in South Africa and the UK show mixed results. Some similar effects on group cohesion are found in these online communities, in particular in Stormfront South Africa, but the effects are smaller in magnitude and less enduring. Specifically, in the case of the South African Tshwane Protests, their effects are maintained for approximately 3 months in the case of members’ attraction to group and for approximately 6 months for unification (whereas for Stormfront Downunder, both these effects are maintained for much longer than 6 months). For the other indicators of group cohesion (bonding and engagement), no significant effects are detected. In the case of Stormfront Britain, coinciding with the UK 2008 election success, there is a short-lived spike in online social interactions in Stormfront Britain, but no longer-term effects are observed in any of the indicators of group cohesion. In both contexts, the events do not seem to produce a significant surge in new, highly active members, thus no group reinvigoration occurs.

All three studies employed identical designs to test the effects of significant socio-political events ‘in real life’ on the group cohesion of online far-right communities. These were based around significant events for each community that were similar (in the sense that they shared at least one of the properties of: ideological alignment to the white supremacist movement in the respective context, and presence of intergroup conflict, collective group goals, and perceived group success). However, our results seem to reflect dynamics that are representative of the different socio-political contexts of Australia, South Africa, and the UK. These differences in the results are useful to identify particular aspects that can determine whether a socio-political event has the potential to become impactful or not. For instance, our findings show that the greatest and most enduring impact was that of the Cronulla Riots in Australia. These effects may be explained by considering the particular significance of the riots in the context of local politics and intergroup relationships at the time. That is, historically, the riots represent the first time in Australia that the debate around national identity explicitly brought strong anti-Muslim attitudes into the public focus. The anti-Muslim sentiment has since become one of the main drivers of the far-right movement in Australia [41], with the actions of the Australian white supremacist responsible for the 2019 Christchurch attacks (who was at least partly radicalised online, 8) being a poignant illustration of this trend. The synergy between the local political trends and the ideological content driving the event (a nascent and growing anti-Muslim sentiment) in the Australian context is not present to the same degree in the South African and British contexts.

Furthermore, while the events in each of the three contexts are underpinned by various degrees of intergroup conflict, open intergroup violence is only present in the case of the Cronulla Riots. This suggests that events that manifest as more extreme forms of collective actions (which are also likely to attract more media attention) have a greater impact on associated online communities. One implication of strong intergroup conflict is that it enables the optimal conditions for increased meta-contrast between opposing groups to occur, so that the outgroup is more directly identifiable from the ingroup’s perspective. These conditions may have, in turn, enabled communications between members of Stormfront Downunder to more effectively drive the process of consensualisation [34] and therefore enable a more robust and functional re-definition of the collective identity of the online community (providing clarity about who they are as a group and who they stand against). In other words, increased group cohesion meant that members strongly bonded under a shared collective (white supremacist) identity where the increasingly strong anti-Muslim focus [32] ensured that the collective identity stayed relevant for a longer period of time. Although similar patterns are observed in the Stormfront South Africa online community, reinvigoration driven by new members (joining immediately after the event) seems unique to the Stormfront Downunder. This suggests that in the Australian context, the Cronulla Riots were more effective in mobilising a social identity highly consistent with norms of far-right activism. Stormfront Downunder provided an ideal outlet for these identities to be enacted and the involvement of highly active new members ensured the long-lasting effects of the riots in the Australian context.

Our findings suggest that local events, which are significant for the far-right movement, may have a pervasive and enduring legacy on local online communities under certain conditions. The differences in our results across the three communities suggest that, such events can be particularly effective when they are more aligned to current trends and attitudes in society and when they represent strong and potentially violent intergroup conflict where the opposite sides are unambiguous. In other words, socio-political events driven by attitudes both towards a group’s ‘enemy’ who is easy to identify (exploiting intergroup conflict) and which are more strongly endorsed by the societal trends at the time (thereby ensuring perceptions of group success) appear to have the greatest effect on the online communities studied. Democratic societies are increasingly threatened by white supremacy and other divisive ideologies that are spread online and energise whole communities of like-minded extremists. To protect the groups that are the most vulnerable from attacks by those affiliated to such extremist online communities, governments need to devise more effective prevention measures–measures which are based on an understanding of what unifies and strengthens online communities linked to political violence.

## Supporting information

S1 Data (DOCX)

S1 Table T-test, H_1_: That average posts per thread per week over the years immediately following the Cronulla Riots (2005–2010) are greater than those over the period 2001–2004 (from the commencement of the sub-forums to the year prior to the riots). (DOCX)

S2 Table T-test, H_1_: That average posts per thread per week over the years immediately following the Tshwane Protests (2005–2010) are greater than those over the period 2001–2004 (from the commencement of the sub-forums to the year prior to the Protests). (DOCX)

S3 Table T-test, H_1_: That, average posts per thread per week over the years immediately following the UK Elections (2008–2013) are greater than those over the period 2004–2007 (baseline being the average of four years prior to the elections). (DOCX)

S4 Table Hypothesis test (t-test, H_1_): That the average number of connections per member post Cronulla Riots was greater than the average over the period prior to the riots for the Stormfront Downunder sub-forum. (DOCX)
