# Supplementary material for: The effects of local socio-political events on group cohesion in online far-right communities
Source: PLoS One. 2020 Mar 30;15(3):e0230302. doi: 10.1371/journal.pone.0230302 (PMC7105128; doi:10.1371/journal.pone.0230302)
Supplement: S1 Data — (DOCX) [file pone.0230302.s001.docx]

**Supplementary Materials**

**Data extraction, creation of summary statistics and graphs**

User post data was collected from the Stormfront Internet forum (https://www.stormfront.org/) from 13/09/2001 to 06/05/2015. The metadata extracted from each post consisted of the unique post identifier (PostID), a user identifier (AuthorID, anonymous users coded as -1), the thread identifier (ThreadID), Unix timestamp, and textual content (which was only used for the analysis of word counts in user posts). Two additional fields were derived from this data for the analysis that followed. These were “Year” and “Window”. Year was derived directly from the Unix timestamp. “Window” refers to the week of the observation. For Stormfront Downunder, the week of the Cronulla Riots was set as Week 0, centered on the first day of the riots, December 11, 2005. For Stormfront South Africa, Week 0 was May 25, 2005, corresponding to the commencement of the Tshwane Protests. For Stormfront Britain, Week 0 was May 1 2008, corresponding to the UK Election. An extract of a typical *Rdata file used in the analysis is shown below.

**PostID ThreadID AuthorID UnixTimestamp PostContent**

**1 8671 1640 110 2001-09-13 19:08:00 Last ...**

**2 10920 2067 1325 2001-09-19 03:02:00 I hav ...**

**3 11204 2121 110 2001-09-19 22:26:00 Ninem ...**

**4 11232 2121 113 2001-09-20 00:05:00 of co ...**

**5 11242 2121 931 2001-09-20 01:09:00 I agr ...**

**6 11281 2121 111 2001-09-20 04:34:00 What' ...**

**7 11305 2067 901 2001-09-20 06:34:00 well ...**

**8 11628 2067 27 2001-09-20 23:06:00 Well ...**

**9 11745 2121 -1 2001-09-21 07:38:00 Quote ...**

**10 13022 2437 111 2001-09-24 05:56:00 A fam ...**

**Bonding**

|   Stormfront South Africa  13 weeks pre- Tshwane Protests |   Stormfront South Africa  Week of Tshwane Protests |   Stormfront South Africa  13 weeks post- Tshwane Protests |
| --- | --- | --- |

**S1 Fig. Social networks formed at 3 months (during Weeks 13) before and after the Tshwane Protests, and during the week of the protests by the 50 most active members over the period from one year prior to one-year post Tshwane Protests.**

|   Stormfront Britain  13 weeks pre- UK elections |   Stormfront Britain  Week of UK elections |   Stormfront Britain  13 weeks post- UK elections |
| --- | --- | --- |

**S2 Fig. Social networks formed at 3 months (during Weeks 13) before and after the UK elections, and during the week of the elections by the 50 most active members over the period from one year prior to one-year post elections.**

**Unification**

**S3 Fig. Posts per thread per week annually, Stormfront South Africa**

**S4 Fig. Posts per thread per week annually, Stormfront Britain**


**Engagement**

**S5 Fig. The number of active members per week before and after Tshwane Protests for Stormfront South Africa by member type (that is, by whether or not the member joined pre- or post- Tshwane Protests).**

**S6 Fig. The number of active members per week before and after UK Elections for Stormfront Britain by member type (that is, by whether or not the member joined pre- or post- UK Elections).**

**Sensitivity to time periods as grouping variable for creation of social networks**


**R Scripts (Stormfront Downunder)**

All analysis was performed in R. The R version used, and platform is given below.

**R version 3.4.1 (2017-06-30) -- "Single Candle"**

**Copyright (C) 2017 The R Foundation for Statistical Computing**

**Platform: x86_64-apple-darwin15.6.0 (64-bit)**

**Analysis of word count per post (anonymous vs identified authors)**

**# Word Count Anonymous and Identified User Posts.R**

**rm(list = ls())**

**library(ggplot2)**

**# library(igraph)**

**# library(plyr)**

**# # # #Downunder Forum**

**# load("SFD.short.RData")**

**# SF = SFD.short**

**# rm(SFD.short)**

**# # #South Africa Forum**

**# load("sfsa.short.RData")**

**# SF = sfsa.short**

**# rm(sfsa.short)**

**# #Britain Forum**

**load("sfuk.short.RData")**

**SF = sfuk.short**

**rm(sfuk.short)**

**#Format Conversions for Britain**

**SF$AuthorID = as.numeric((SF$AuthorID))**

**SF$PostID = as.numeric((SF$PostID))**

**SF$ThreadID = as.numeric((SF$ThreadID))**

**SF$UnixTimestamp = as.POSIXct((SF$UnixTimestamp))**

**SF$newdate = strptime(SF$UnixTimestamp, format = "%Y-%m-%d")**

**dd = SF[c(1,2,3,5)]**

**# dd = dd[1:10,]**

**dd$Wordcount = lengths(gregexpr("\\W+", dd$PostContent)) + 1**

**WCidentified = dd[!(dd$AuthorID== -1),]**

**WCidentified = WCidentified[complete.cases(WCidentified), ]**

**WCanon = dd[(dd$AuthorID== -1),]**

**WCanon = WCanon[complete.cases(WCanon), ]**

**mean(WCidentified$Wordcount)**

**sd(WCidentified$Wordcount)**

**mean(WCanon$Wordcount)**

**sd(WCanon$Wordcount)**

**#Standardised Mean Difference**

**(mean(WCanon$Wordcount) - mean(WCidentified$Wordcount))/sd(c(dd$Wordcount))**

**# t.test(WCanon$Wordcount,WCidentified$Wordcount, "t")**

**# t.test(WCanon$Wordcount,WCidentified$Wordcount, "t", var.equal = TRUE)**

**summary(WCidentified$Wordcount)**

**summary(WCanon$Wordcount)**

**# t.test(log10(WCanon$Wordcount),log10(WCidentified$Wordcount), "t", var.equal = TRUE)**

**t.test(log10(WCanon$Wordcount),log10(WCidentified$Wordcount), "t")**

**Figure 1**

**rm(list = ls())**

**SFMeta = read.csv("SFrontDownunderMeta.csv")**

**attach(SFMeta)**

**Posts = as.data.frame(table(Window))**

**# count all unique threads per week**

**Thread = as.data.frame(as.table(by(ThreadID, Window, FUN = function(x) length(unique(x)))))**

**# count all unique authors per week**

**Author = as.data.frame(as.table(by(AuthorID, Window, FUN = function(x) length(unique(x)))))**

**# now index by year**

**Year = as.data.frame(as.table(trunc(by(Year, Window, mean))))**

**PAT = cbind(Posts, Author, Thread, Year)**

**PAT = PAT[,c(1,2,4,6, 8)]**

**colnames(PAT) = c("Week", "Posts", "Authors", "Threads", "Year" )**

**# calculate posts per thread**

**PBT = PAT$Posts/PAT$Threads**

**PAT = cbind(PAT, PBT)**

**rm(PBT)**

**write.csv(PAT, "PATWeek.csv", row.names = FALSE)**

**library(ggplot2)**

**# dotplot of posts per week**

**PPperWK <- ggplot(data = PAT, aes(Week, Posts)) + ylim(0,1000) + theme_bw() + ylab("Posts per week") + xlab("Year")**

**PPperWK <- PPperWK + geom_point(shape = 20, colour = "black", size = 3)**

**PPperWK <- PPperWK + scale_x_discrete(breaks = c(-205, -153, -101, -49, 4, 56, 108, 160, 213, 265, 317, 369, 421, 473), labels = c("2002", "2003", "2004", "2005", "2006", "2007", "2008", "2009", "2010", "2011", "2012", "2013", "2014", "2015") )**

**PPperWK = PPperWK + ggtitle("Number of Posts per Week: Stormfront Downunder forum")**

**PPperWK**

**ggsave("PPperWK.pdf", PPperWK, width = 10, height = 6, unit = "in")**

**ggsave("PPperWK.eps", PPperWK, width = 10, height = 6, unit = "in")**

**# to do a boxplot of posts per thread by year**

**PBThread = ggplot(PAT, aes(factor(Year), PBT)) + geom_boxplot() + theme_bw() + xlab("Years") + ylab("Posts Per Thread") + ylim(0,20) + ggtitle("Posts per Thread per Week")**

**PBThread**

**ggsave("PBThread.pdf", PBThread, width = 10, height = 6, unit = "in")**

**# annual summary of posts and post per thread**

**YSUM = as.data.frame(table(PAT$Year))**

**YMean = as.data.frame(as.table(tapply(PAT$Posts, PAT$Year, mean)))**

**YSD = as.data.frame(as.table(tapply(PAT$Posts, PAT$Year, sd)))**

**YPBTMean = as.data.frame(as.table(tapply(PAT$PBT, PAT$Year, mean)))**

**YPBTSD = as.data.frame(as.table(tapply(PAT$PBT, PAT$Year, sd)))**

**YSUM = cbind(YSUM, YMean, YSD, YPBTMean, YPBTSD)**

**YSUM = YSUM[,c(1,2,4,6,8,10)]**

**colnames(YSUM) = c("Year", "N", "Mean", "SD", "PBT Mean", "PBT SD")**

**YSD = NULL**

**YMean = NULL**

**YPBTMean = NULL**

**YPBTSD = NULL**

**write.csv(YSUM, "YSUM.csv", row.names = FALSE)**

**Figure 2, Hypothesis Tests for activity pre- post- event, Correlation between forums**

**# For PLOS review.R**

**# This graphs the activity time series plots all 3 sub-forums**

**# and calculates all correlations between time series at significant events**

**# and performs hypothesis tests of activity pre- post- significant event**

**rm(list = ls())**

**library(ggplot2)**

**# 'Weeks' of arbitrary number of days pre/post riot**

**weeklength = 7**

**midweek = floor(weeklength/2)**

**#Checked this is putting midweek in the right spot**

**#Downunder Forum**

**load("SFD.short.RData")**

**SF = SFD.short**

**rm(SFD.short)**

**# Days pre/post riot**

**SF$newdate = as.Date(SF$UnixTimestamp, format = "%Y-%m-%d")**

**SF$riotdays = as.Date(SF$newdate, format = "%Y-%m-%d") - as.Date("2005-12-11", format = "%Y-%m-%d")**

**SF$week = floor((SF$riotdays + midweek)/weeklength)**

**SF = SF[c(1,2,3,6,7,8)]**

**#Summary Stormfront Downunder**

**summary(SF)**

**#Anonymous posts**

**Anon = subset(SF, (AuthorID == "-1"))**

**length(Anon$PostID)**

**Posts.D = as.data.frame(table(SF$week))**

**colnames(Posts.D) = c("week", "posts")**

**#South Africa Forum**

**load("sfsa.short.RData")**

**SF = sfsa.short**

**rm(sfsa.short)**

**# Days pre/post riot**

**SF$newdate = as.Date(SF$UnixTimestamp, format = "%Y-%m-%d")**

**SF$riotdays = as.Date(SF$newdate, format = "%Y-%m-%d") - as.Date("2005-12-11", format = "%Y-%m-%d")**

**SF$week = floor((SF$riotdays + midweek)/weeklength)**

**SF = SF[c(1,2,3,6,7,8)]**

**#Summary Stormfront South Africa**

**summary(SF)**

**#Anonymous posts**

**Anon = subset(SF, (AuthorID == "-1"))**

**length(Anon$PostID)**

**Posts.SA = as.data.frame(table(SF$week))**

**colnames(Posts.SA) = c("week", "posts")**

**SumPost <- merge(Posts.D, Posts.SA, by="week", all.x = TRUE, all.y = TRUE, sort = TRUE)**

**colnames(SumPost) = c("week", "SFD","SFSA")**

**#United Kingdom Forum**

**load("sfuk.short.RData")**

**SF = sfuk.short**

**rm(sfuk.short)**

**# Days pre/post riot**

**SF$newdate = as.Date(SF$UnixTimestamp, format = "%Y-%m-%d")**

**SF$riotdays = as.Date(SF$newdate, format = "%Y-%m-%d") - as.Date("2005-12-11", format = "%Y-%m-%d")**

**SF$week = floor((SF$riotdays + midweek)/weeklength)**

**SF = SF[c(1,2,3,6,7,8)]**

**#Summary Stormfront Britain**

**summary(SF)**

**#Anonymous posts**

**Anon = subset(SF, (AuthorID == "-1"))**

**length(Anon$PostID)**

**Posts.UK = as.data.frame(table(SF$week))**

**colnames(Posts.UK) = c("week", "posts")**

**SumPost <- merge(SumPost, Posts.UK, by="week", all.x = TRUE, all.y = TRUE, sort = TRUE)**

**SumPost <- SumPost[order(SumPost$week),]**

**colnames(SumPost) = c("week", "SFD","SFSA", "SFUK")**

**write.csv(SumPost, "SumPost.csv", row.names = FALSE)**

**XX = read.csv("SumPost.csv")**

**XX <- XX[order(XX$week),]**

**# XX <- subset(XX, ( (week < 158) & (week > -105)))**

**#**

**g = ggplot(XX, aes(x = week)) +**

**geom_line(aes(y = SFD), colour="blue") +**

**geom_line(aes(y = SFSA), colour = "red") +**

**geom_line(aes(y = SFUK), colour = "darkgreen") +**

**annotate("text", label = "Stormfront Britain", x = -100, y = 2000, size = 3, colour = "darkgreen", hjust = "inward") +**

**annotate("text", label = "Stormfront Downunder", x = -100, y = 550, size = 3, colour = "blue", hjust = "inward") +**

**annotate("text", label = "Stormfront South Africa", x = -100, y = 400, size = 3, colour = "red", hjust = "inward") +**

**geom_vline(xintercept = 0) +**

**annotate("text", label = "Cronulla Riots", x = 4, y = 4000, size = 3, hjust = "inward", colour = "blue") +**

**geom_vline(xintercept = -28) +**

**annotate("text", label = "Tshwane Protests", x = -65, y = 4000, size = 3, hjust = "inward", colour = "red") +**

**geom_vline(xintercept = 124) +**

**annotate("text", label = "UK Local Elections", x = 128, y = 4000, size = 3, hjust = "outward", colour = "darkgreen") +**

**ylab(label="Posts per Week") +**

**xlab("Week")**

**g**

**ggsave("ForumCompareAll.pdf", g, width = 10, height = 6, unit = "in")**

**PPWlow = subset(XX, ( (week >= -26) & (week <= -1)))**

**PPWhigh = subset(XX, ( (week >= 2) & (week <= 27)))**

**t.test(PPWhigh$SFD,PPWlow$SFD, alternative = "greater")**

**# additional stats**

**mean(PPWlow$SFD)**

**sd(PPWlow$SFD)**

**mean(PPWhigh$SFD)**

**sd(PPWhigh$SFD)**

**#Standardised Mean Difference**

**(mean(PPWhigh$SFD) - mean(PPWlow$SFD))/sd(c(PPWhigh$SFD,PPWlow$SFD))**

**# TTests for significant activity around Tshwane Protests**

**# Stormfront South Africa**

**# TTests replication around Tshwane Riots (not significant for SA @ 26 weeks)**

**# Protest Weeks -29 and -28**

**# Interval is 26 Weeks**

**PPWlow = subset(XX, ( (week >= -55) & (week <= -30)))**

**PPWhigh = subset(XX, ( (week >= -27) & (week <= -2)))**

**t.test(PPWhigh$SFSA,PPWlow$SFSA, alternative = "greater")**

**# additional stats**

**mean(PPWlow$SFSA)**

**sd(PPWlow$SFSA)**

**mean(PPWhigh$SFSA)**

**sd(PPWhigh$SFSA)**

**#Standardised Mean Difference**

**(mean(PPWhigh$SFSA) - mean(PPWlow$SFSA))/sd(c(PPWhigh$SFSA,PPWlow$SFSA))**

**# TTests replication around Tshwane Riots (significant for SA @ 13 weeks)**

**# Interval is 13 Weeks**

**PPWlow = subset(XX, ( (week >= -42) & (week <= -30)))**

**PPWhigh = subset(XX, ( (week >= -27) & (week <= -15)))**

**t.test(PPWhigh$SFSA,PPWlow$SFSA, alternative = "greater")**

**# additional stats**

**mean(PPWlow$SFSA)**

**sd(PPWlow$SFSA)**

**mean(PPWhigh$SFSA)**

**sd(PPWhigh$SFSA)**

**#Standardised Mean Difference**

**(mean(PPWhigh$SFSA) - mean(PPWlow$SFSA))/sd(c(PPWhigh$SFSA,PPWlow$SFSA))**

**# TTests replication around UK Elections @ 26 weeks)**

**# Weeks 124 and 125**

**PPWlow = subset(XX, ( (week >= 98) & (week <= 123)))**

**PPWhigh = subset(XX, ( (week >= 126) & (week <= 151)))**

**t.test(PPWhigh$SFUK,PPWlow$SFUK, alternative = "greater")**

**# additional stats**

**mean(PPWlow$SFUK)**

**sd(PPWlow$SFUK)**

**mean(PPWhigh$SFUK)**

**sd(PPWhigh$SFUK)**

**#Standardised Mean Difference**

**(mean(PPWhigh$SFUK) - mean(PPWlow$SFUK))/sd(c(PPWhigh$SFUK,PPWlow$SFUK))**

**# TTests replication around UK Elections @ 13 weeks)**

**# Weeks 124 and 125**

**PPWlow = subset(XX, ( (week >= 111) & (week <= 123)))**

**PPWhigh = subset(XX, ( (week >= 126) & (week <= 138)))**

**t.test(PPWhigh$SFUK,PPWlow$SFUK, alternative = "greater")**

**# additional stats**

**mean(PPWlow$SFUK)**

**sd(PPWlow$SFUK)**

**mean(PPWhigh$SFUK)**

**sd(PPWhigh$SFUK)**

**#Standardised Mean Difference**

**(mean(PPWhigh$SFUK) - mean(PPWlow$SFUK))/sd(c(PPWhigh$SFUK,PPWlow$SFUK))**

**Figure 3**

**rm(list = ls())**

**SFMeta = read.csv("SFrontDownunderMeta.csv")**

**library(igraph)**

**library(plyr)**

**# delete unused columns**

**dd = SFMeta [,c(1,3,4,5)]**

**# delete anonymous**

**dd<-dd[!(dd$AuthorID== -1),]**

**# init any data frames for rbind here**

**weekdf = data.frame()**

**edgedf = data.frame()**

**vertexdf = data.frame()**

**# now do the rest week by week**

**for (theweek in -105:105){**

**ddx <- subset(dd, ( (Window == theweek)))**

**# now only keep ddx authors in topNusers**

**#ddx = ddx[(ddx$AuthorID %in% topNusers$AuthorID),]**

**# count threads and delete any with n = 1**

**nthreads = table(ddx$ThreadID)**

**nthreads = sort(nthreads, decreasing = T)**

**nthreads = as.data.frame(nthreads)**

**nthreads = nthreads[!(nthreads$Freq==1),]**

**#nthreads = as.data.frame(nthreads)**

**colnames(nthreads) = c("ThreadID", "Freq")**

**# now only keep threads with more than one post**

**ddx = ddx[(ddx$ThreadID %in% nthreads$ThreadID),]**

**# now delete unused columns and duplicate thread/author pairs**

**ddx = ddx[,c(3,4)]**

**ddx = unique(ddx)**

**ddx = ddx[order(ddx$ThreadID),]**

**# now recount nthreads**

**nthreads = table(ddx$ThreadID)**

**nthreads = sort(nthreads, decreasing = T)**

**nthreads = as.data.frame(nthreads)**

**nthreads = nthreads[!(nthreads$Freq==1),]**

**colnames(nthreads) = c("ThreadID", "Freq")**

**autpairs = data.frame(Aut1=numeric(),Aut2=numeric())**

**for (x in 1:nrow(nthreads))**

**{**

**ddy <- subset(ddx, ( (ThreadID == nthreads$ThreadID[x])))**

**#print(ddy$AuthorID)**

**tempautpairs = as.data.frame(t(combn(ddy$AuthorID,2)))**

**#print(autpairs)**

**autpairs = rbind(autpairs, tempautpairs)**

**}**

**g = graph_from_data_frame(autpairs, FALSE,NULL)**

**# create adj matrix and sort it**

**gdetail = as_adjacency_matrix(g, type = "both", attr = NULL, edges = FALSE, names = TRUE, sparse = FALSE)**

**gdetail = as.data.frame(gdetail)**

**gdetail$index <- as.numeric(row.names(gdetail))**

**gdetail = gdetail[order(gdetail$index),]**

**gdetail$index = NULL**

**gdetail = as.matrix(gdetail)**

**# transpose and repeat**

**ngdetail = t(gdetail)**

**ngdetail = as.data.frame(ngdetail)**

**ngdetail$index <- as.numeric(row.names(ngdetail))**

**ngdetail = ngdetail[order(ngdetail$index),]**

**ngdetail$index = NULL**

**ngdetail = as.matrix(ngdetail)**

**# print(paste("VEA", theweek, vcount(g), ecount(g), format(average.path.length(g), digits = 4)))**

**weekdf = rbind(weekdf, theweek)**

**edgedf = rbind(edgedf, ecount(g))**

**vertexdf = rbind(vertexdf, vcount(g))**

**} # theweek**

**Wplotdata = cbind(weekdf,vertexdf,edgedf,edgedf/vertexdf)**

**colnames(Wplotdata) = c("Week","Threads","Members","ThreadsMem")**

**write.csv(Wplotdata, "Wplotdata.csv", row.names = FALSE)**

**library(ggplot2)**

**g = qplot(Week, ThreadsMem, data = Wplotdata) + geom_line() + ylim(0,25)**

**g = g + ylab("Members") + theme_bw()**

**#g = g + xlim(-100,100)**

**g = g + xlim(-52,52)**

**g = g + ggtitle("Connections per Member")**

**g**

**ggsave("APL ConnMembX.pdf", g, width = 10, height = 6, unit = "in")**

**Hypothesis Tests, Attraction of Individual to the group**

**#Attraction Network Calcs (rdata) All Members All Forums V03.R**

**#This performs all network calculations all subforums comprising:**

**#Hypothesis testing**

**#Sensitivity analysis**

**# Have to choose (a) Forum, (b) event date, (c) number of weeks to hyp test**

**rm(list = ls())**

**library(ggplot2)**

**library(igraph)**

**library(plyr)**

**savetreport <- data.frame()**

**# # #Downunder Forum**

**# load("SFD.short.RData")**

**# SF = SFD.short**

**# rm(SFD.short)**

**# # #South Africa Forum**

**# load("sfsa.short.RData")**

**# SF = sfsa.short**

**# rm(sfsa.short)**

**# #Britain Forum**

**load("sfuk.short.RData")**

**SF = sfuk.short**

**rm(sfuk.short)**

**SF$newdate = strptime(SF$UnixTimestamp, format = "%Y-%m-%d")**

**## Automated function runs from here**

**## Put variable required for sensitivity analysis below**

**for (STartfinish in c(13)){**

**for (WEeklength in c(7)){**

**for (NTopusers in c(10000)){**

**#-----------------------------------------**

**# 'Weeks' of arbitrary number of days pre/post riot**

**#WEeklength = 7**

**midweek = floor(WEeklength/2)**

**# Choose the significant event and center "week"**

**#SF$riotdays = as.Date(SF$newdate, format = "%Y-%m-%d") - as.Date("2005-12-11", format = "%Y-%m-%d")**

**# Cronulla Riots**

**# SF$riotdays = as.Date(SF$newdate, format = "%Y-%m-%d") - as.Date("2005-12-11", format = "%Y-%m-%d")**

**# Tshwane Riots**

**# use 28/5 for SA**

**# SF$riotdays = as.Date(SF$newdate, format = "%Y-%m-%d") - as.Date("2005-05-25", format = "%Y-%m-%d")**

**# UK Election**

**SF$riotdays = as.Date(SF$newdate, format = "%Y-%m-%d") - as.Date("2008-05-1", format = "%Y-%m-%d")**

**SF$Window = floor((SF$riotdays + midweek)/WEeklength)**

**SFX = SF[c(1,8,2,3,4,6)]**

**#write.csv(SF, "Test Meta B.csv", row.names = FALSE)**

**# delete unused columns**

**dd = SFX**

**dd<-dd[!(dd$AuthorID== -1),]**

**# weeks pre- post- Cronulla to use**

**dd <- subset(dd, ( (Window <= (STartfinish + 1)) & (Window >= -STartfinish)))**

**# next code makes list of top N (50) authors**

**topNusers <- table(dd$AuthorID)**

**topNusers = sort(topNusers, decreasing = T)**

**topNusers = head(topNusers, NTopusers)**

**# identifying labels**

**newlabs = c(1:NTopusers)**

**topNusers = as.data.frame(topNusers)**

**colnames(topNusers) = c("AuthorID", "Freq")**

**# init output dataframes**

**saveout <- data.frame()**

**savelow <- data.frame()**

**savehigh <- data.frame()**

**# now do the rest week by week**

**for (theweek in c(-STartfinish:(STartfinish + 1))){**

**ddx <- subset(dd, ( (Window == theweek)))**

**# now only keep ddx authors in topNusers**

**ddx = ddx[(ddx$AuthorID %in% topNusers$AuthorID),]**

**# count threads and delete any with n = 1**

**nthreads = table(ddx$ThreadID)**

**nthreads = sort(nthreads, decreasing = T)**

**nthreads = as.data.frame(nthreads)**

**nthreads = nthreads[!(nthreads$Freq==1),]**

**#nthreads = as.data.frame(nthreads)**

**colnames(nthreads) = c("ThreadID", "Freq")**

**# now only keep threads with more than one post**

**ddx = ddx[(ddx$ThreadID %in% nthreads$ThreadID),]**

**# now delete unused columns and duplicate thread/author pairs**

**ddx = ddx[,c(3,4)]**

**ddx = unique(ddx)**

**ddx = ddx[order(ddx$ThreadID),]**

**# now recount nthreads**

**nthreads = table(ddx$ThreadID)**

**nthreads = sort(nthreads, decreasing = T)**

**nthreads = as.data.frame(nthreads)**

**nthreads = nthreads[!(nthreads$Freq==1),]**

**colnames(nthreads) = c("ThreadID", "Freq")**

**autpairs = data.frame(Aut1=numeric(),Aut2=numeric())**

**for (x in 1:nrow(nthreads))**

**{**

**ddy <- subset(ddx, ( (ThreadID == nthreads$ThreadID[x])))**

**#print(ddy$AuthorID)**

**tempautpairs = as.data.frame(t(combn(ddy$AuthorID,2)))**

**#print(autpairs)**

**autpairs = rbind(autpairs, tempautpairs)**

**}**

**g = graph_from_data_frame(autpairs, FALSE,NULL)**

**# create adj matrix and sort it**

**gdetail = as_adjacency_matrix(g, type = "both", attr = NULL, edges = FALSE, names = TRUE, sparse = FALSE)**

**gdetail = as.data.frame(gdetail)**

**gdetail$index <- as.numeric(row.names(gdetail))**

**gdetail = gdetail[order(gdetail$index),]**

**gdetail$index = NULL**

**gdetail = as.matrix(gdetail)**

**# transpose and repeat**

**ngdetail = t(gdetail)**

**ngdetail = as.data.frame(ngdetail)**

**ngdetail$index <- as.numeric(row.names(ngdetail))**

**ngdetail = ngdetail[order(ngdetail$index),]**

**ngdetail$index = NULL**

**ngdetail = as.matrix(ngdetail)**

**gg = graph_from_adjacency_matrix(ngdetail, mode = "undirected", weighted = NULL, diag = TRUE, add.rownames = TRUE)**

**ConnMemb = format(ecount(gg)/vcount(gg), digits = 4)**

**print(paste("VEA", STartfinish, WEeklength, theweek, vcount(gg), ecount(gg), ConnMemb, format(average.path.length(gg), digits = 4)))**

**savetemp <- c(theweek, vcount(gg), ecount(gg), as.numeric(ConnMemb) )**

**if (theweek < 0) {savelow <- rbind(savelow, savetemp)}**

**if (theweek > 1) {savehigh <- rbind(savehigh, savetemp)}**

**saveout <- rbind(saveout, savetemp)**

**} # theweek**

**colnames(saveout) = c("Week", "Vertices","Edges", "ConnMemb")**

**colnames(savelow) = c("Week", "Vertices","Edges", "ConnMemb")**

**colnames(savehigh) = c("Week", "Vertices","Edges", "ConnMemb")**

**filename = paste("Sum W",WEeklength,"N",NTopusers,"D",STartfinish,".csv", collapse = "")**

**## write.csv(saveout, filename, row.names = FALSE)**

**t.test(savehigh$ConnMemb,savelow$ConnMemb, alternative = "greater")**

**ttp = t.test(savehigh$ConnMemb,savelow$ConnMemb, alternative = "greater")$p.value**

**ttstatistic = t.test(savehigh$ConnMemb,savelow$ConnMemb, alternative = "greater")$statistic**

**ttdof = t.test(savehigh$ConnMemb,savelow$ConnMemb, alternative = "greater")$parameter**

**ttdom = t.test(savehigh$ConnMemb,savelow$ConnMemb, alternative = "greater")$estimate**

**meanhigh = mean(savehigh$ConnMemb)**

**sdhigh = sd(savehigh$ConnMemb)**

**meanlow = mean(savelow$ConnMemb)**

**sdlow = sd(savelow$ConnMemb)**

**sdiff = (meanhigh - meanlow)/sd(c(savehigh$ConnMemb,savelow$ConnMemb))**

**TOtaldays = WEeklength * STartfinish**

**savetreporttemp <- c(TOtaldays, WEeklength, NTopusers, STartfinish, ttp, ttstatistic, ttdof,**

**meanhigh, sdhigh, meanlow, sdlow, sdiff)**

**savetreport <- rbind(savetreport, savetreporttemp)**

**#-----------------------------------------------------------------**

**## Automated function runs to here**

**} # NOptuser**

**} # WEeklength**

**} # STartfinish**

**colnames(savetreport) = c("Days", "Week","TopMemb", "S-F", "pValue", "tStat", "DOF",**

**"meanhigh", "sdhigh", "meanlow", "sdlow", "sdiff")**

**write.csv(savetreport, "HypTest Report PLOS SFUK Local Elections 3 Months.csv", row.names = FALSE)**

**Figure 4 and Network plots all sub-forums**

**rm(list = ls())**

**library(ggplot2)**

**library(igraph)**

**library(plyr)**

**savetreport <- data.frame()**

**# #Downunder Forum**

**load("SFD.short.RData")**

**SF = SFD.short**

**rm(SFD.short)**

**# # #South Africa Forum**

**# load("sfsa.short.RData")**

**# SF = sfsa.short**

**# rm(sfsa.short)**

**# # #Britain Forum**

**# load("sfuk.short.RData")**

**# SF = sfuk.short**

**# rm(sfuk.short)**

**SF$newdate = strptime(SF$UnixTimestamp, format = "%Y-%m-%d")**

**## Automated function runs from here**

**## Put variable required for sensitivity analysis below**

**STartfinish = 26**

**WEeklength = 7**

**NTopusers = 50**

**#-----------------------------------------**

**# 'Weeks' of arbitrary number of days pre/post riot**

**#WEeklength = 7**

**midweek = floor(WEeklength/2)**

**#check this is putting midweek in the right spot**

**# Choose the significant event and center "week"**

**#SF$riotdays = as.Date(SF$newdate, format = "%Y-%m-%d") - as.Date("2005-12-11", format = "%Y-%m-%d")**

**# Cronulla Riots**

**SF$riotdays = as.Date(SF$newdate, format = "%Y-%m-%d") - as.Date("2005-12-11", format = "%Y-%m-%d")**

**# Fake Day - small effect**

**# SF$riotdays = as.Date(SF$newdate, format = "%Y-%m-%d") - as.Date("2011-12-11", format = "%Y-%m-%d")**

**# Tshwane Riots**

**# use 28/5 for SA**

**# SF$riotdays = as.Date(SF$newdate, format = "%Y-%m-%d") - as.Date("2005-05-25", format = "%Y-%m-%d")**

**# UK Election**

**# SF$riotdays = as.Date(SF$newdate, format = "%Y-%m-%d") - as.Date("2008-05-1", format = "%Y-%m-%d")**

**SF$Window = floor((SF$riotdays + midweek)/WEeklength)**

**SFX = SF[c(1,8,2,3,4,6)]**

**#write.csv(SF, "Test Meta B.csv", row.names = FALSE)**

**# delete unused columns**

**dd = SFX**

**#-----------------------------------------------------------------**

**# 'Weeks' of arbitrary number of days pre/post riot default, == 7**

**## WEeklength = 7**

**#midweek = floor(WEeklength/2)**

**#check this is putting midweek in the right spot**

**# number of top users, default == 50**

**## NTopusers = 200**

**# start and finish weeks, default == 52**

**#STartfinish = 52**

**#STartfinish = STartfinish + 1**

**#Downunder Forum**

**# Days pre/post riot**

**#SFMeta$newdate = as.Date(SFMeta$UnixTimestamp, format = "%Y-%m-%d")**

**#SFMeta$riotdays = as.Date(SFMeta$newdate, format = "%Y-%m-%d") - as.Date("2005-12-11", format = "%Y-%m-%d")**

**# SFMeta$week = floor((SFMeta$riotdays + midweek)/WEeklength)**

**# use "Window" to corresond to older code**

**#SFMeta$Window = floor((SFMeta$riotdays + midweek)/WEeklength)**

**# Tested to confirm that weeklength 7 and original date settings convert unix timestamp**

**# to correspond to our original analysis. Therefore day 0 = 0 and weeks the same.**

**# delete unused columns**

**#dd = SFMeta [,c(1,3,4,5)]**

**dd<-dd[!(dd$AuthorID== -1),]**

**# weeks pre- post- Cronulla to use**

**dd <- subset(dd, ( (Window <= (STartfinish + 1)) & (Window >= -STartfinish)))**

**# next code makes list of top N (50) authors**

**topNusers <- table(dd$AuthorID)**

**topNusers = sort(topNusers, decreasing = T)**

**topNusers = head(topNusers, NTopusers)**

**# identifying labels**

**newlabs = c(1:NTopusers)**

**topNusers = as.data.frame(topNusers)**

**colnames(topNusers) = c("AuthorID", "Freq")**

**# init any data frames for rbind here**

**# saveout <- data.frame(degx = numeric(), edgex = numeric())**

**saveout <- data.frame()**

**savelow <- data.frame()**

**savehigh <- data.frame()**

**# now do the rest week by week**

**# for (theweek in -52:52){**

**for (theweek in c(-STartfinish:(STartfinish + 1))){**

**ddx <- subset(dd, ( (Window == theweek)))**

**# now only keep ddx authors in topNusers**

**ddx = ddx[(ddx$AuthorID %in% topNusers$AuthorID),]**

**# count threads and delete any with n = 1**

**nthreads = table(ddx$ThreadID)**

**nthreads = sort(nthreads, decreasing = T)**

**nthreads = as.data.frame(nthreads)**

**nthreads = nthreads[!(nthreads$Freq==1),]**

**#nthreads = as.data.frame(nthreads)**

**colnames(nthreads) = c("ThreadID", "Freq")**

**# now only keep threads with more than one post**

**ddx = ddx[(ddx$ThreadID %in% nthreads$ThreadID),]**

**# now delete unused columns and duplicate thread/author pairs**

**ddx = ddx[,c(3,4)]**

**ddx = unique(ddx)**

**ddx = ddx[order(ddx$ThreadID),]**

**# now recount nthreads**

**nthreads = table(ddx$ThreadID)**

**nthreads = sort(nthreads, decreasing = T)**

**nthreads = as.data.frame(nthreads)**

**nthreads = nthreads[!(nthreads$Freq==1),]**

**colnames(nthreads) = c("ThreadID", "Freq")**

**autpairs = data.frame(Aut1=numeric(),Aut2=numeric())**

**for (x in 1:nrow(nthreads))**

**{**

**ddy <- subset(ddx, ( (ThreadID == nthreads$ThreadID[x])))**

**#print(ddy$AuthorID)**

**tempautpairs = as.data.frame(t(combn(ddy$AuthorID,2)))**

**#print(autpairs)**

**autpairs = rbind(autpairs, tempautpairs)**

**}**

**g = graph_from_data_frame(autpairs, FALSE,NULL)**

**# get authors not in original graph**

**ingraph = V(g)$name**

**ingraph = as.data.frame(ingraph)**

**extras = ddx = topNusers[(!topNusers$AuthorID %in% ingraph$ingraph),]**

**if (nrow(extras) > 0) {**

**for (x in 1:nrow(extras))**

**{**

**g <- add_vertices(g, 1, name = as.character(extras$AuthorID[x]))**

**}**

**}**

**# create adj matrix and sort it**

**gdetail = as_adjacency_matrix(g, type = "both", attr = NULL, edges = FALSE, names = TRUE, sparse = FALSE)**

**gdetail = as.data.frame(gdetail)**

**gdetail$index <- as.numeric(row.names(gdetail))**

**gdetail = gdetail[order(gdetail$index),]**

**gdetail$index = NULL**

**gdetail = as.matrix(gdetail)**

**# transpose and repeat**

**ngdetail = t(gdetail)**

**ngdetail = as.data.frame(ngdetail)**

**ngdetail$index <- as.numeric(row.names(ngdetail))**

**ngdetail = ngdetail[order(ngdetail$index),]**

**ngdetail$index = NULL**

**ngdetail = as.matrix(ngdetail)**

**gg = graph_from_adjacency_matrix(ngdetail, mode = "undirected", weighted = NULL, diag = TRUE, add.rownames = TRUE)**

**plot(gg, layout = layout.circle, edge.color = "grey", vertex.color = "white", vertex.label = newlabs, main = theweek)**

**# pdf("plot.pdf", 10, 10)**

**printweek = theweek + 1000**

**pdf(sprintf("Week %s.pdf", printweek), 10, 10)**

**#igraph.options(plot.layout = layout.circle, edge.color = "grey", vertex.color = "white", vertex.label = newlabs, vertex.size = 10)**

**igraph.options(plot.layout = layout.circle, vertex.color = "white", vertex.label = newlabs, vertex.size = 10, main = theweek)**

**plot(gg)**

**dev.off()**

**print(paste("VEA", theweek, vcount(gg), ecount(gg), format(average.path.length(gg), digits = 4)))**

**ConnMemb = format(ecount(gg)/vcount(gg), digits = 4)**

**print(paste("VEA", theweek, vcount(gg), ecount(gg), ConnMemb, format(average.path.length(gg), digits = 4)))**

**} # theweek**

**#-----------------------------------------------------------------**

**## Automated function runs to here**

**Figure 5**

**rm(list = ls())**

**SFMeta = read.csv("SFrontDownunderMeta.csv")**

**library(igraph)**

**library(plyr)**

**# delete unused columns**

**dd = SFMeta [,c(1,3,4,5)]**

**dd<-dd[!(dd$AuthorID== -1),]**

**# weeks pre- post- Cronulla to use**

**dd <- subset(dd, ( (Window < 53) & (Window > -53)))**

**# next code makes list of top N (50) authors**

**topNusers <- table(dd$AuthorID)**

**topNusers = sort(topNusers, decreasing = T)**

**topNusers = head(topNusers, 50)**

**# identifying labels**

**newlabs = c(1:50)**

**topNusers = as.data.frame(topNusers)**

**colnames(topNusers) = c("AuthorID", "Freq")**

**# init any data frames for rbind here**

**# now do the rest week by week**

**heatdata = data.frame()**

**for (theweek in -52:52){**

**ddx <- subset(dd, ( (Window == theweek)))**

**# now only keep ddx authors in topNusers**

**ddx = ddx[(ddx$AuthorID %in% topNusers$AuthorID),]**

**# count threads and delete any with n = 1**

**nthreads = table(ddx$ThreadID)**

**nthreads = sort(nthreads, decreasing = T)**

**nthreads = as.data.frame(nthreads)**

**nthreads = nthreads[!(nthreads$Freq==1),]**

**#nthreads = as.data.frame(nthreads)**

**colnames(nthreads) = c("ThreadID", "Freq")**

**# now only keep threads with more than one post**

**ddx = ddx[(ddx$ThreadID %in% nthreads$ThreadID),]**

**# now delete unused columns and duplicate thread/author pairs**

**ddx = ddx[,c(3,4)]**

**ddx = unique(ddx)**

**ddx = ddx[order(ddx$ThreadID),]**

**# now recount nthreads**

**nthreads = table(ddx$ThreadID)**

**nthreads = sort(nthreads, decreasing = T)**

**nthreads = as.data.frame(nthreads)**

**nthreads = nthreads[!(nthreads$Freq==1),]**

**colnames(nthreads) = c("ThreadID", "Freq")**

**autpairs = data.frame(Aut1=numeric(),Aut2=numeric())**

**for (x in 1:nrow(nthreads))**

**{**

**ddy <- subset(ddx, ( (ThreadID == nthreads$ThreadID[x])))**

**#print(ddy$AuthorID)**

**tempautpairs = as.data.frame(t(combn(ddy$AuthorID,2)))**

**#print(autpairs)**

**autpairs = rbind(autpairs, tempautpairs)**

**}**

**g = graph_from_data_frame(autpairs, FALSE,NULL)**

**# get authors not in original graph**

**ingraph = V(g)$name**

**ingraph = as.data.frame(ingraph)**

**extras = ddx = topNusers[(!topNusers$AuthorID %in% ingraph$ingraph),]**

**if (nrow(extras) > 0) {**

**for (x in 1:nrow(extras))**

**{**

**g <- add_vertices(g, 1, name = as.character(extras$AuthorID[x]))**

**}**

**}**

**# create adj matrix and sort it**

**gdetail = as_adjacency_matrix(g, type = "both", attr = NULL, edges = FALSE, names = TRUE, sparse = FALSE)**

**gdetail = as.data.frame(gdetail)**

**gdetail$index <- as.numeric(row.names(gdetail))**

**gdetail = gdetail[order(gdetail$index),]**

**gdetail$index = NULL**

**gdetail = as.matrix(gdetail)**

**# transpose and repeat**

**ngdetail = t(gdetail)**

**ngdetail = as.data.frame(ngdetail)**

**ngdetail$index <- as.numeric(row.names(ngdetail))**

**ngdetail = ngdetail[order(ngdetail$index),]**

**ngdetail$index = NULL**

**ngdetail = as.matrix(ngdetail)**

**rdetailsums = as.data.frame(as.table(colSums(ngdetail)))**

**rdetailsums = rdetailsums[,2]**

**heatdata = rbind(heatdata, t(rdetailsums))**

**# this creates a file for the individual week.**

**# write.csv(ngdetail, "ngdetail.csv", row.names = TRUE)**

**gg = graph_from_adjacency_matrix(ngdetail, mode = "undirected", weighted = NULL, diag = TRUE, add.rownames = TRUE)**

**#gg = graph_from_adjacency_matrix(ngdetail, mode = "undirected", weighted = TRUE, diag = TRUE, add.rownames = TRUE)**

**## plot(gg, layout = layout.circle, vertex.color = "red", vertex.label = newlabs)**

**# pdf("plot.pdf", 10, 10)**

**#pdf(sprintf("Week %s.pdf", theweek), 10, 10)**

**#igraph.options(plot.layout = layout.circle, vertex.color = "red", vertex.label = newlabs, vertex.size = 10)**

**#plot(gg)**

**#dev.off()**

**} # theweek**

**heatdata = t(heatdata)**

**write.csv(heatdata, "heatdata.csv", row.names = TRUE)**

**my_palette <- colorRampPalette(c("red", "yellow"))(n = 256)**

**g = heatmap(heatdata, Rowv=NA, Colv=NA, col=my_palette, scale="column", main, na.rm = TRUE, verbose = getOption("verbose"))**

**g**

**Figure 7 and Hypothesis tests for unification**

**#Unification PPT V01.R**

**#This performs all posts per thread calculations for all subforums**

**rm(list = ls())**

**library(ggplot2)**

**# 'Weeks' of arbitrary number of days pre/post riot**

**weeklength = 7**

**midweek = floor(weeklength/2)**

**#Checked this is putting midweek in the right spot**

**#Downunder Forum**

**load("SFD.short.RData")**

**SF = SFD.short**

**rm(SFD.short)**

**# Days pre/post riot**

**SF$newdate = strptime(SF$UnixTimestamp, format = "%Y-%m-%d")**

**# SF$newdate = as.Date(SF$UnixTimestamp, format = "%Y-%m-%d")**

**SF$riotdays = as.Date(SF$newdate, format = "%Y-%m-%d") - as.Date("2005-12-11", format = "%Y-%m-%d")**

**SF$week = floor((SF$riotdays + midweek)/weeklength)**

**SF = SF[c(1,2,3,6,7,8)]**

**#Summary Stormfront Downunder**

**summary(SF)**

**#Anonymous posts**

**Anon = subset(SF, (AuthorID == "-1"))**

**length(Anon$PostID)**

**Posts.D = as.data.frame(table(SF$week))**

**Threads.D = as.data.frame(as.table(by(SF$ThreadID, SF$week, FUN = function(x) length(unique(x)))))**

**colnames(Posts.D) = c("week", "posts")**

**colnames(Threads.D) = c("week", "threads")**

**SumPost <- merge(Posts.D, Threads.D, by="week", all.x = TRUE, all.y = TRUE, sort = TRUE)**

**colnames(SumPost) = c("week", "SFD","SFDT")**

**#South Africa Forum**

**load("sfsa.short.RData")**

**SF = sfsa.short**

**rm(sfsa.short)**

**# Days pre/post riot**

**SF$newdate = strptime(SF$UnixTimestamp, format = "%Y-%m-%d")**

**# SF$newdate = as.Date(SF$UnixTimestamp, format = "%Y-%m-%d")**

**SF$riotdays = as.Date(SF$newdate, format = "%Y-%m-%d") - as.Date("2005-12-11", format = "%Y-%m-%d")**

**SF$week = floor((SF$riotdays + midweek)/weeklength)**

**SF = SF[c(1,2,3,6,7,8)]**

**#Summary Stormfront South Africa**

**summary(SF)**

**#Anonymous posts**

**Anon = subset(SF, (AuthorID == "-1"))**

**length(Anon$PostID)**

**Posts.SA = as.data.frame(table(SF$week))**

**Threads.SA = as.data.frame(as.table(by(SF$ThreadID, SF$week, FUN = function(x) length(unique(x)))))**

**colnames(Posts.SA) = c("week", "posts")**

**colnames(Threads.SA) = c("week", "threads")**

**SumPost <- merge(SumPost, Posts.SA, by="week", all.x = TRUE, all.y = TRUE, sort = TRUE)**

**SumPost <- merge(SumPost, Threads.SA, by="week", all.x = TRUE, all.y = TRUE, sort = TRUE)**

**colnames(SumPost) = c("week", "SFD","SFDT", "SFSA", "SFSAT")**

**#United Kingdom Forum**

**load("sfuk.short.RData")**

**SF = sfuk.short**

**rm(sfuk.short)**

**#Format Conversions for Britain**

**SF$AuthorID = as.numeric((SF$AuthorID))**

**SF$PostID = as.numeric((SF$PostID))**

**SF$ThreadID = as.numeric((SF$ThreadID))**

**SF$UnixTimestamp = as.POSIXct((SF$UnixTimestamp))**

**# Days pre/post riot**

**SF$newdate = strptime(SF$UnixTimestamp, format = "%Y-%m-%d")**

**# SF$newdate = as.Date(SF$UnixTimestamp, format = "%Y-%m-%d")**

**SF$riotdays = as.Date(SF$newdate, format = "%Y-%m-%d") - as.Date("2005-12-11", format = "%Y-%m-%d")**

**SF$week = floor((SF$riotdays + midweek)/weeklength)**

**SF = SF[c(1,2,3,6,7,8)]**

**#Summary Stormfront Britain**

**summary(SF)**

**#Anonymous posts**

**Anon = subset(SF, (AuthorID == "-1"))**

**length(Anon$PostID)**

**Posts.UK = as.data.frame(table(SF$week))**

**Threads.UK = as.data.frame(as.table(by(SF$ThreadID, SF$week, FUN = function(x) length(unique(x)))))**

**SF$Year = format(as.Date(SF$newdate, format="%d/%m/%Y"),"%Y")**

**SF$Year = as.numeric(SF$Year)**

**Year.UK = as.data.frame(as.table(by(SF$Year, SF$week, FUN = min)))**

**colnames(Posts.UK) = c("week", "posts")**

**colnames(Threads.UK) = c("week", "threads")**

**colnames(Year.UK) = c("week", "year")**

**SumPost <- merge(SumPost, Posts.UK, by="week", all.x = TRUE, all.y = TRUE, sort = TRUE)**

**SumPost <- merge(SumPost, Threads.UK, by="week", all.x = TRUE, all.y = TRUE, sort = TRUE)**

**SumPost <- merge(SumPost, Year.UK, by="week", all.x = TRUE, all.y = TRUE, sort = TRUE)**

**colnames(SumPost) = c("week", "SFD","SFDT", "SFSA", "SFSAT", "SFUK", "SFUKT", "Year")**

**SumPost <- SumPost[order(SumPost$week),]**

**write.csv(SumPost, "SumPostPBT.csv", row.names = FALSE)**

**XX = read.csv("SumPostPBT.csv")**

**XX <- XX[order(XX$week),]**

**XX<-XX[(XX$Year < 2016),]**

**XX$DPBT = XX$SFD/XX$SFDT**

**# to do a boxplot of posts per thread by year**

**PBThread = ggplot(XX, aes(factor(Year), DPBT)) + geom_boxplot() + theme_bw() + xlab("Years") + ylab("Posts Per Thread") + ylim(0,20) + ggtitle("Posts per Thread per Week")**

**PBThread**

**ggsave("PBThread Downunder.pdf", PBThread, width = 10, height = 6, unit = "in")**

**XX$SAPBT = XX$SFSA/XX$SFSAT**

**# to do a boxplot of posts per thread by year**

**PBThread = ggplot(XX, aes(factor(Year), SAPBT)) + geom_boxplot() + theme_bw() + xlab("Years") + ylab("Posts Per Thread") + ylim(0,20) + ggtitle("Posts per Thread per Week")**

**PBThread**

**ggsave("PBThread South Africa.pdf", PBThread, width = 10, height = 6, unit = "in")**

**XX$UKPBT = XX$SFUK/XX$SFUKT**

**# to do a boxplot of posts per thread by year**

**PBThread = ggplot(XX, aes(factor(Year), UKPBT)) + geom_boxplot() + theme_bw() + xlab("Years") + ylab("Posts Per Thread") + ylim(0,20) + ggtitle("Posts per Thread per Week")**

**PBThread**

**ggsave("PBThread Britain.pdf", PBThread, width = 10, height = 6, unit = "in")**

**# Hypothesis Tests Downunder**

**rm(SF)**

**rm(Posts.D,Posts.SA,Posts.UK)**

**rm(Threads.D,Threads.SA,Threads.UK,Year.UK)**

**rm(Anon,SumPost)**

**library(car)**

**resulttable = data.frame()**

**XX$TYear = recode(XX$Year, " '2001' = '2004'; '2002' = '2004'; '2003' = '2004'")**

**PBTpre = subset(XX, XX$TYear == "2004")**

**PBTpost = subset(XX, XX$TYear == "2005")**

**PBTpre = PBTpre[complete.cases(PBTpre), ]**

**PBTpost = PBTpost[complete.cases(PBTpost), ]**

**ttest = t.test(PBTpost$DPBT,PBTpre$DPBT, alternative = "greater", var.equal=TRUE)**

**d = (mean(PBTpost$DPBT) - mean(PBTpre$DPBT))/sd(c(PBTpost$DPBT,PBTpre$DPBT))**

**resultsx = c("SD", "2005", mean(PBTpre$DPBT),sd(PBTpre$DPBT),mean(PBTpost$DPBT),sd(PBTpost$DPBT),**

**ttest$parameter,ttest$statistic,ttest$p.value,d)**

**resultsx = as.data.frame(t(resultsx))**

**resulttable = rbind(resulttable, resultsx)**

**PBTpre = subset(XX, XX$TYear == "2004")**

**PBTpost = subset(XX, XX$TYear == "2006")**

**PBTpre = PBTpre[complete.cases(PBTpre), ]**

**PBTpost = PBTpost[complete.cases(PBTpost), ]**

**ttest = t.test(PBTpost$DPBT,PBTpre$DPBT, alternative = "greater", var.equal=TRUE)**

**d = (mean(PBTpost$DPBT) - mean(PBTpre$DPBT))/sd(c(PBTpost$DPBT,PBTpre$DPBT))**

**resultsx = c("SD", "2006", mean(PBTpre$DPBT),sd(PBTpre$DPBT),mean(PBTpost$DPBT),sd(PBTpost$DPBT),**

**ttest$parameter,ttest$statistic,ttest$p.value,d)**

**resultsx = as.data.frame(t(resultsx))**

**resulttable = rbind(resulttable, resultsx)**

**PBTpre = subset(XX, XX$TYear == "2004")**

**PBTpost = subset(XX, XX$TYear == "2007")**

**PBTpre = PBTpre[complete.cases(PBTpre), ]**

**PBTpost = PBTpost[complete.cases(PBTpost), ]**

**ttest = t.test(PBTpost$DPBT,PBTpre$DPBT, alternative = "greater", var.equal=TRUE)**

**d = (mean(PBTpost$DPBT) - mean(PBTpre$DPBT))/sd(c(PBTpost$DPBT,PBTpre$DPBT))**

**resultsx = c("SD", "2007", mean(PBTpre$DPBT),sd(PBTpre$DPBT),mean(PBTpost$DPBT),sd(PBTpost$DPBT),**

**ttest$parameter,ttest$statistic,ttest$p.value,d)**

**resultsx = as.data.frame(t(resultsx))**

**resulttable = rbind(resulttable, resultsx)**

**PBTpre = subset(XX, XX$TYear == "2004")**

**PBTpost = subset(XX, XX$TYear == "2008")**

**PBTpre = PBTpre[complete.cases(PBTpre), ]**

**PBTpost = PBTpost[complete.cases(PBTpost), ]**

**ttest = t.test(PBTpost$DPBT,PBTpre$DPBT, alternative = "greater", var.equal=TRUE)**

**d = (mean(PBTpost$DPBT) - mean(PBTpre$DPBT))/sd(c(PBTpost$DPBT,PBTpre$DPBT))**

**resultsx = c("SD", "2008", mean(PBTpre$DPBT),sd(PBTpre$DPBT),mean(PBTpost$DPBT),sd(PBTpost$DPBT),**

**ttest$parameter,ttest$statistic,ttest$p.value,d)**

**resultsx = as.data.frame(t(resultsx))**

**resulttable = rbind(resulttable, resultsx)**

**PBTpre = subset(XX, XX$TYear == "2004")**

**PBTpost = subset(XX, XX$TYear == "2009")**

**PBTpre = PBTpre[complete.cases(PBTpre), ]**

**PBTpost = PBTpost[complete.cases(PBTpost), ]**

**ttest = t.test(PBTpost$DPBT,PBTpre$DPBT, alternative = "greater", var.equal=TRUE)**

**d = (mean(PBTpost$DPBT) - mean(PBTpre$DPBT))/sd(c(PBTpost$DPBT,PBTpre$DPBT))**

**resultsx = c("SD", "2009", mean(PBTpre$DPBT),sd(PBTpre$DPBT),mean(PBTpost$DPBT),sd(PBTpost$DPBT),**

**ttest$parameter,ttest$statistic,ttest$p.value,d)**

**resultsx = as.data.frame(t(resultsx))**

**resulttable = rbind(resulttable, resultsx)**

**PBTpre = subset(XX, XX$TYear == "2004")**

**PBTpost = subset(XX, XX$TYear == "2010")**

**PBTpre = PBTpre[complete.cases(PBTpre), ]**

**PBTpost = PBTpost[complete.cases(PBTpost), ]**

**ttest = t.test(PBTpost$DPBT,PBTpre$DPBT, alternative = "greater", var.equal=TRUE)**

**d = (mean(PBTpost$DPBT) - mean(PBTpre$DPBT))/sd(c(PBTpost$DPBT,PBTpre$DPBT))**

**resultsx = c("SD", "2010", mean(PBTpre$DPBT),sd(PBTpre$DPBT),mean(PBTpost$DPBT),sd(PBTpost$DPBT),**

**ttest$parameter,ttest$statistic,ttest$p.value,d)**

**resultsx = as.data.frame(t(resultsx))**

**resulttable = rbind(resulttable, resultsx)**

**# Hypothesis Tests South Africa**

**PBTpre = subset(XX, XX$TYear == "2004")**

**PBTpost = subset(XX, XX$TYear == "2005")**

**PBTpre = PBTpre[complete.cases(PBTpre), ]**

**PBTpost = PBTpost[complete.cases(PBTpost), ]**

**ttest = t.test(PBTpost$SAPBT,PBTpre$SAPBT, alternative = "greater", var.equal=TRUE)**

**d = (mean(PBTpost$SAPBT) - mean(PBTpre$SAPBT))/sd(c(PBTpost$SAPBT,PBTpre$SAPBT))**

**resultsx = c("SSA", "2005", mean(PBTpre$SAPBT),sd(PBTpre$SAPBT),mean(PBTpost$SAPBT),sd(PBTpost$SAPBT),**

**ttest$parameter,ttest$statistic,ttest$p.value,d)**

**resultsx = as.data.frame(t(resultsx))**

**resulttable = rbind(resulttable, resultsx)**

**PBTpre = subset(XX, XX$TYear == "2004")**

**PBTpost = subset(XX, XX$TYear == "2006")**

**PBTpre = PBTpre[complete.cases(PBTpre), ]**

**PBTpost = PBTpost[complete.cases(PBTpost), ]**

**ttest = t.test(PBTpost$SAPBT,PBTpre$SAPBT, alternative = "greater", var.equal=TRUE)**

**d = (mean(PBTpost$SAPBT) - mean(PBTpre$SAPBT))/sd(c(PBTpost$SAPBT,PBTpre$SAPBT))**

**resultsx = c("SSA", "2006", mean(PBTpre$SAPBT),sd(PBTpre$SAPBT),mean(PBTpost$SAPBT),sd(PBTpost$SAPBT),**

**ttest$parameter,ttest$statistic,ttest$p.value,d)**

**resultsx = as.data.frame(t(resultsx))**

**resulttable = rbind(resulttable, resultsx)**

**PBTpre = subset(XX, XX$TYear == "2004")**

**PBTpost = subset(XX, XX$TYear == "2007")**

**PBTpre = PBTpre[complete.cases(PBTpre), ]**

**PBTpost = PBTpost[complete.cases(PBTpost), ]**

**ttest = t.test(PBTpost$SAPBT,PBTpre$SAPBT, alternative = "greater", var.equal=TRUE)**

**d = (mean(PBTpost$SAPBT) - mean(PBTpre$SAPBT))/sd(c(PBTpost$SAPBT,PBTpre$SAPBT))**

**resultsx = c("SSA", "2007", mean(PBTpre$SAPBT),sd(PBTpre$SAPBT),mean(PBTpost$SAPBT),sd(PBTpost$SAPBT),**

**ttest$parameter,ttest$statistic,ttest$p.value,d)**

**resultsx = as.data.frame(t(resultsx))**

**resulttable = rbind(resulttable, resultsx)**

**PBTpre = subset(XX, XX$TYear == "2004")**

**PBTpost = subset(XX, XX$TYear == "2008")**

**PBTpre = PBTpre[complete.cases(PBTpre), ]**

**PBTpost = PBTpost[complete.cases(PBTpost), ]**

**ttest = t.test(PBTpost$SAPBT,PBTpre$SAPBT, alternative = "greater", var.equal=TRUE)**

**d = (mean(PBTpost$SAPBT) - mean(PBTpre$SAPBT))/sd(c(PBTpost$SAPBT,PBTpre$SAPBT))**

**resultsx = c("SSA", "2008", mean(PBTpre$SAPBT),sd(PBTpre$SAPBT),mean(PBTpost$SAPBT),sd(PBTpost$SAPBT),**

**ttest$parameter,ttest$statistic,ttest$p.value,d)**

**resultsx = as.data.frame(t(resultsx))**

**resulttable = rbind(resulttable, resultsx)**

**PBTpre = subset(XX, XX$TYear == "2004")**

**PBTpost = subset(XX, XX$TYear == "2009")**

**PBTpre = PBTpre[complete.cases(PBTpre), ]**

**PBTpost = PBTpost[complete.cases(PBTpost), ]**

**ttest = t.test(PBTpost$SAPBT,PBTpre$SAPBT, alternative = "greater", var.equal=TRUE)**

**d = (mean(PBTpost$SAPBT) - mean(PBTpre$SAPBT))/sd(c(PBTpost$SAPBT,PBTpre$SAPBT))**

**resultsx = c("SSA", "2009", mean(PBTpre$SAPBT),sd(PBTpre$SAPBT),mean(PBTpost$SAPBT),sd(PBTpost$SAPBT),**

**ttest$parameter,ttest$statistic,ttest$p.value,d)**

**resultsx = as.data.frame(t(resultsx))**

**resulttable = rbind(resulttable, resultsx)**

**PBTpre = subset(XX, XX$TYear == "2004")**

**PBTpost = subset(XX, XX$TYear == "2010")**

**PBTpre = PBTpre[complete.cases(PBTpre), ]**

**PBTpost = PBTpost[complete.cases(PBTpost), ]**

**ttest = t.test(PBTpost$SAPBT,PBTpre$SAPBT, alternative = "greater", var.equal=TRUE)**

**d = (mean(PBTpost$SAPBT) - mean(PBTpre$SAPBT))/sd(c(PBTpost$SAPBT,PBTpre$SAPBT))**

**resultsx = c("SSA", "2010", mean(PBTpre$SAPBT),sd(PBTpre$SAPBT),mean(PBTpost$SAPBT),sd(PBTpost$SAPBT),**

**ttest$parameter,ttest$statistic,ttest$p.value,d)**

**resultsx = as.data.frame(t(resultsx))**

**resulttable = rbind(resulttable, resultsx)**

**# # Hypothesis Tests Britain**

**#**

**# Just use 4 years pre-elections (same as for Downunder and South Africa)**

**#XX$TYear = recode(XX$Year, " '2001' = '2007'; '2002' = '2007'; '2003' = '2007'; '2004' = '2007'; '2005' = '2007'; '2006' = '2007'")**

**XX$TYear = recode(XX$Year, " '2004' = '2007'; '2005' = '2007'; '2006' = '2007'")**

**PBTpre = subset(XX, XX$TYear == "2007")**

**PBTpost = subset(XX, XX$TYear == "2008")**

**PBTpre = PBTpre[complete.cases(PBTpre), ]**

**PBTpost = PBTpost[complete.cases(PBTpost), ]**

**ttest = t.test(PBTpost$UKPBT,PBTpre$UKPBT, alternative = "greater", var.equal=TRUE)**

**d = (mean(PBTpost$UKPBT) - mean(PBTpre$UKPBT))/sd(c(PBTpost$UKPBT,PBTpre$UKPBT))**

**resultsx = c("SUK", "2008", mean(PBTpre$UKPBT),sd(PBTpre$UKPBT),mean(PBTpost$UKPBT),sd(PBTpost$UKPBT),**

**ttest$parameter,ttest$statistic,ttest$p.value,d)**

**resultsx = as.data.frame(t(resultsx))**

**resulttable = rbind(resulttable, resultsx)**

**PBTpre = subset(XX, XX$TYear == "2007")**

**PBTpost = subset(XX, XX$TYear == "2009")**

**PBTpre = PBTpre[complete.cases(PBTpre), ]**

**PBTpost = PBTpost[complete.cases(PBTpost), ]**

**ttest = t.test(PBTpost$UKPBT,PBTpre$UKPBT, alternative = "greater", var.equal=TRUE)**

**d = (mean(PBTpost$UKPBT) - mean(PBTpre$UKPBT))/sd(c(PBTpost$UKPBT,PBTpre$UKPBT))**

**resultsx = c("SUK", "2009", mean(PBTpre$UKPBT),sd(PBTpre$UKPBT),mean(PBTpost$UKPBT),sd(PBTpost$UKPBT),**

**ttest$parameter,ttest$statistic,ttest$p.value,d)**

**resultsx = as.data.frame(t(resultsx))**

**resulttable = rbind(resulttable, resultsx)**

**PBTpre = subset(XX, XX$TYear == "2007")**

**PBTpost = subset(XX, XX$TYear == "2010")**

**PBTpre = PBTpre[complete.cases(PBTpre), ]**

**PBTpost = PBTpost[complete.cases(PBTpost), ]**

**ttest = t.test(PBTpost$UKPBT,PBTpre$UKPBT, alternative = "greater", var.equal=TRUE)**

**d = (mean(PBTpost$UKPBT) - mean(PBTpre$UKPBT))/sd(c(PBTpost$UKPBT,PBTpre$UKPBT))**

**resultsx = c("SUK", "2010", mean(PBTpre$UKPBT),sd(PBTpre$UKPBT),mean(PBTpost$UKPBT),sd(PBTpost$UKPBT),**

**ttest$parameter,ttest$statistic,ttest$p.value,d)**

**resultsx = as.data.frame(t(resultsx))**

**resulttable = rbind(resulttable, resultsx)**

**PBTpre = subset(XX, XX$TYear == "2007")**

**PBTpost = subset(XX, XX$TYear == "2011")**

**PBTpre = PBTpre[complete.cases(PBTpre), ]**

**PBTpost = PBTpost[complete.cases(PBTpost), ]**

**ttest = t.test(PBTpost$UKPBT,PBTpre$UKPBT, alternative = "greater", var.equal=TRUE)**

**d = (mean(PBTpost$UKPBT) - mean(PBTpre$UKPBT))/sd(c(PBTpost$UKPBT,PBTpre$UKPBT))**

**resultsx = c("SUK", "2011", mean(PBTpre$UKPBT),sd(PBTpre$UKPBT),mean(PBTpost$UKPBT),sd(PBTpost$UKPBT),**

**ttest$parameter,ttest$statistic,ttest$p.value,d)**

**resultsx = as.data.frame(t(resultsx))**

**resulttable = rbind(resulttable, resultsx)**

**PBTpre = subset(XX, XX$TYear == "2007")**

**PBTpost = subset(XX, XX$TYear == "2012")**

**PBTpre = PBTpre[complete.cases(PBTpre), ]**

**PBTpost = PBTpost[complete.cases(PBTpost), ]**

**ttest = t.test(PBTpost$UKPBT,PBTpre$UKPBT, alternative = "greater", var.equal=TRUE)**

**d = (mean(PBTpost$UKPBT) - mean(PBTpre$UKPBT))/sd(c(PBTpost$UKPBT,PBTpre$UKPBT))**

**resultsx = c("SUK", "2012", mean(PBTpre$UKPBT),sd(PBTpre$UKPBT),mean(PBTpost$UKPBT),sd(PBTpost$UKPBT),**

**ttest$parameter,ttest$statistic,ttest$p.value,d)**

**resultsx = as.data.frame(t(resultsx))**

**resulttable = rbind(resulttable, resultsx)**

**PBTpre = subset(XX, XX$TYear == "2007")**

**PBTpost = subset(XX, XX$TYear == "2013")**

**PBTpre = PBTpre[complete.cases(PBTpre), ]**

**PBTpost = PBTpost[complete.cases(PBTpost), ]**

**ttest = t.test(PBTpost$UKPBT,PBTpre$UKPBT, alternative = "greater", var.equal=TRUE)**

**d = (mean(PBTpost$UKPBT) - mean(PBTpre$UKPBT))/sd(c(PBTpost$UKPBT,PBTpre$UKPBT))**

**resultsx = c("SUK", "2013", mean(PBTpre$UKPBT),sd(PBTpre$UKPBT),mean(PBTpost$UKPBT),sd(PBTpost$UKPBT),**

**ttest$parameter,ttest$statistic,ttest$p.value,d)**

**resultsx = as.data.frame(t(resultsx))**

**resulttable = rbind(resulttable, resultsx)**

**colnames(resulttable) = c("Forum", "Year", "Mean Pre", "SD Pre", "Mean Post", "SD Post", "DF", "t", "p.value", "d")**

**write.csv(resulttable, "HypTest Report PLOS Unification all forums.csv", row.names = FALSE)**

**Figure 7**

**# Engagement Pre-Post RData V00.R**

**# This creates the user posting activity pre- post- significant event**

**rm(list = ls())**

**library(ggplot2)**

**# library(igraph)**

**# library(plyr)**

**# # #Downunder Forum**

**load("SFD.short.RData")**

**SF = SFD.short**

**# rm(SFD.short)**

**# # #South Africa Forum**

**# load("sfsa.short.RData")**

**# SF = sfsa.short**

**# rm(sfsa.short)**

**# # #Britain Forum**

**# load("sfuk.short.RData")**

**# SF = sfuk.short**

**# rm(sfuk.short)**

**#Format Conversions for Britain**

**SF$AuthorID = as.numeric((SF$AuthorID))**

**SF$PostID = as.numeric((SF$PostID))**

**SF$ThreadID = as.numeric((SF$ThreadID))**

**SF$UnixTimestamp = as.POSIXct((SF$UnixTimestamp))**

**SF$newdate = strptime(SF$UnixTimestamp, format = "%Y-%m-%d")**

**WEeklength = 7**

**midweek = floor(WEeklength/2)**

**# Cronulla Riots**

**#SF$riotdays = as.Date(SF$newdate, format = "%Y-%m-%d") - as.Date("2005-12-11", format = "%Y-%m-%d")**

**# Tshwane Riots**

**# SF$riotdays = as.Date(SF$newdate, format = "%Y-%m-%d") - as.Date("2005-05-25", format = "%Y-%m-%d")**

**# UK Election**

**SF$riotdays = as.Date(SF$newdate, format = "%Y-%m-%d") - as.Date("2008-05-1", format = "%Y-%m-%d")**

**SF$Window = floor((SF$riotdays + midweek)/WEeklength)**

**dd = SF[c(1,8,2,3)]**

**#-----------------------------------------------------------------**

**offset = 0**

**dd<-dd[!(dd$AuthorID== -1),]**

**# Original Windows**

**#dd <- subset(dd, ( (Window < 105) & (Window > -105)))**

**#ee <- subset(dd, ( (Window < 0) & (Window > -105)))**

**dd <- subset(dd, ( (Window < 105 + offset) & (Window > -105 + offset)))**

**ee <- subset(dd, ( (Window < 0 + offset) & (Window > -105 + offset)))**

**# want posts by author in weeks leading up to riot ee**

**attach(ee)**

**Posts = as.data.frame(as.**

**(by(PostID, AuthorID, FUN = function(x) length(unique(x)))))**

**# median(Posts$Freq)**

**# mean(Posts$Freq)**

**# Posts$activepre <- ifelse(Posts$Freq > 2, c("High"), c("Low"))**

**Posts$activepre <- ifelse(Posts$Freq > 1, c("Existing"), c("Existing"))**

**dd$active = Posts$activepre[match(as.character(dd$AuthorID), as.character(Posts$AuthorID))]**

**dd[is.na(dd)] = "New"**

**postsweek = as.data.frame(as.table(tapply(dd$PostID, list(dd$Window, dd$active), length)))**

**# count the number of authors posting each week**

**authorsweek = as.data.frame(as.table(tapply(dd$AuthorID, list(dd$Window, dd$active), FUN = function(x) length(unique(x)))))**

**colnames(authorsweek) = c("Week", "Status", "NActive")**

**g = qplot(Week, NActive, data = authorsweek, group =Status, color = Status, geom = "path")**

**g = g + ylim(0,150)**

**# g = g + geom_point(size = 0.01)**

**g = g + ylab("Active members") + theme_bw()**

**g = g + scale_x_discrete(breaks=seq(-200,240,20))**

**g = g + ggtitle("Number of Active Members per week by Member Type")**

**g = g + scale_colour_manual(values = c("darkgray", "black"))**

**g = g + geom_vline(xintercept = 0)**

**g**

**ggsave("MemberActivity Pre-Post X.pdf", g, width = 10, height = 6, unit = "in")**
