# Supplementary material for: The effects of local socio-political events on group cohesion in online far-right communities
Source: PLoS One. 2020 Mar 30;15(3):e0230302. doi: 10.1371/journal.pone.0230302 (PMC7105128; doi:10.1371/journal.pone.0230302)
Supplement: S1 Table — (DOCX) [file pone.0230302.s002.docx]

**S1 Table. T-test, H_1_: that average posts per thread per week over the years immediately following the Cronulla Riots (2005 – 2010) are greater than those over the period 2001 – 2004 (from the commencement of the sub-forums to the year prior to the riots)**

| **Forum** | **Year** | **Mean Pre** | **SD Pre** | **Mean Post** | **SD Post** | **DOF** | **t** | **p.value** | **d** |
| --- | --- | --- | --- | --- | --- | --- | --- | --- | --- |
| SD | 2005 | 5.08 | 3.34 | 8.33 | 2.28 | 218 | 6.559 | 0.000 | 0.953 |
| SD | 2006 | 5.08 | 3.34 | 7.47 | 1.57 | 218 | 4.987 | 0.000 | 0.752 |
| SD | 2007 | 5.08 | 3.34 | 6.35 | 1.71 | 218 | 2.624 | 0.005 | 0.411 |
| SD | 2008 | 5.08 | 3.34 | 5.76 | 1.42 | 218 | 1.424 | 0.078 | 0.226 |
| SD | 2009 | 5.08 | 3.34 | 5.65 | 2.46 | 219 | 1.140 | 0.128 | 0.179 |
| SD | 2010 | 5.08 | 3.34 | 4.54 | 1.07 | 218 | -1.147 | 0.874 | -0.182 |
