# Supplementary material for: The effects of local socio-political events on group cohesion in online far-right communities
Source: PLoS One. 2020 Mar 30;15(3):e0230302. doi: 10.1371/journal.pone.0230302 (PMC7105128; doi:10.1371/journal.pone.0230302)
Supplement: S2 Table — (DOCX) [file pone.0230302.s003.docx]

**S2 Table. T-test, H_1_: that average posts per thread per week over the years immediately following the Tshwane Protests (2005 – 2010) are greater than those over the period 2001 – 2004 (from the commencement of the sub-forums to the year prior to the Protests)**

| **Forum** | **Year** | **Mean Pre** | **SD Pre** | **Mean Post** | **SD Post** | **DOF** | **t** | **p.value** | **d** |
| --- | --- | --- | --- | --- | --- | --- | --- | --- | --- |
| SSA | 2005 | 3.55 | 1.55 | 5.91 | 1.97 | 218 | 8.961 | 0.000 | 1.218 |
| SSA | 2006 | 3.55 | 1.55 | 5.22 | 1.47 | 218 | 6.869 | 0.000 | 0.991 |
| SSA | 2007 | 3.55 | 1.55 | 5.49 | 1.73 | 218 | 7.663 | 0.000 | 1.082 |
| SSA | 2008 | 3.55 | 1.55 | 4.56 | 1.18 | 218 | 4.307 | 0.000 | 0.658 |
| SSA | 2009 | 3.55 | 1.55 | 6.45 | 2.16 | 219 | 10.724 | 0.000 | 1.371 |
| SSA | 2010 | 3.55 | 1.55 | 5.29 | 1.98 | 218 | 6.583 | 0.000 | 0.956 |
