# Supplementary material for: The effects of local socio-political events on group cohesion in online far-right communities
Source: PLoS One. 2020 Mar 30;15(3):e0230302. doi: 10.1371/journal.pone.0230302 (PMC7105128; doi:10.1371/journal.pone.0230302)
Supplement: S3 Table — (DOCX) [file pone.0230302.s004.docx]

**S3 Table. T-test, H_1_: that, average posts per thread per week over the years immediately following the UK Elections (2008 – 2013) are greater than those over the period 2004 – 2007 (baseline being the average of four years prior to the elections)**

| **Forum** | **Year** | **Mean Pre** | **SD Pre** | **Mean Post** | **SD Post** | **DOF** | **t** | **p.value** | **d** |
| --- | --- | --- | --- | --- | --- | --- | --- | --- | --- |
| SUK | 2008 | 7.03 | 1.34 | 8.53 | 1.30 | 259 | 7.254 | 0.000 | 1.027 |
| SUK | 2009 | 7.03 | 1.34 | 6.99 | 2.14 | 260 | -0.166 | 0.566 | -0.026 |
| SUK | 2010 | 7.03 | 1.34 | 6.33 | 1.18 | 259 | -3.423 | 1.000 | -0.520 |
| SUK | 2011 | 7.03 | 1.34 | 5.66 | 0.89 | 259 | -6.966 | 1.000 | -0.993 |
| SUK | 2012 | 7.03 | 1.34 | 6.22 | 0.87 | 259 | -4.131 | 1.000 | -0.621 |
| SUK | 2013 | 7.03 | 1.34 | 6.04 | 0.93 | 259 | -5.027 | 1.000 | -0.745 |
