# Supplementary material for: The effects of local socio-political events on group cohesion in online far-right communities
Source: PLoS One. 2020 Mar 30;15(3):e0230302. doi: 10.1371/journal.pone.0230302 (PMC7105128; doi:10.1371/journal.pone.0230302)
Supplement: S4 Table — (DOCX) [file pone.0230302.s005.docx]

**S4 Table. Hypothesis test (t-test, H_1_): that the average number of connections per member post Cronulla Riots was greater than the average over the period prior to the riots for the Stormfront Downunder sub-forum.**

The number of days over which members posting to the same thread were connected is varied, as is the number of groups used to form the time interval (pre- and post- riots) tested. These results indicate that varying these parameters does affect the terms in the hypothesis test calculation, but that the conclusion drawn is quite insensitive to these parameter variations.

| **Days/Group** | **Groups pre/post** | **MPre** | **MPost** | **t =** | **p-value** | **Sig <0.01** |
| --- | --- | --- | --- | --- | --- | --- |
| 3 | 26 | 5.180 | 6.672 | 2.219 | 0.0155 |  |
| 7 | 26 | 8.496 | 10.387 | 2.532 | 0.0074 | * |
| 10 | 26 | 9.618 | 12.201 | 2.961 | 0.0024 | * |
| 14 | 26 | 11.575 | 13.947 | 2.503 | 0.0078 | * |
| 3 | 52 | 4.952 | 6.499 | 3.373 | 0.0005 | * |
| 7 | 52 | 7.761 | 9.268 | 2.603 | 0.0053 | * |
| 10 | 52 | 8.192 | 11.102 | 4.258 | 0.0000 | * |
| 14 | 52 | 8.965 | 11.984 | 3.861 | 0.0001 | * |
